# Blood Plasma Circulating DNA-Protein Complexes: Involvement in Carcinogenesis and Prospects for Liquid Biopsy of Breast Cancer

**DOI:** 10.3390/jpm13121691

**Published:** 2023-12-05

**Authors:** Aleksei Shefer, Oleg Tutanov, Maxim Belenikin, Yuri P. Tsentalovich, Svetlana Tamkovich

**Affiliations:** 1 V. Zelman Institute for Medicine and Psychology, Novosibirsk State University, 630090 Novosibirsk, Russia; a.shefer@g.nsu.ru; 2 Department of Medicine, Vanderbilt University Medical Center, Nashville, TN 37203, USA; oleg.s.tutanov@vumc.org; 3 Evogen LLC, 115191 Moscow, Russia; 4 International Tomography Center, Siberian Branch of Russian Academy of Sciences, 630090 Novosibirsk, Russia

**Keywords:** circulating DNA, nucleoprotein complexes, plasma, MALDI-TOF mass spectrometry, breast cancer, bioinformatics analysis, liquid biopsy

## Abstract

Circulating DNA (cirDNA) is a promising tool in translational medicine. However, studies of cirDNA have neglected its association with proteins, despite ample evidence that this interaction may affect the fate of DNA in the bloodstream and its molecular functions. The goal of the current study is to shed light on the differences between the proteomic cargos of histone-containing nucleoprotein complexes (NPCs) from healthy female (HFs) and breast cancer patients (BCPs), and to reveal the proteins involved in carcinogenesis. NPCs were isolated from the blood samples of HFs and BCPs using affinity chromatography. A total of 177 and 169 proteins were identified in NPCs from HFs and BCPs using MALDI-TOF mass spectrometry. A bioinformatics analysis revealed that catalytically active proteins, as well as proteins that bind nucleic acids and regulate the activity of receptors, are the most represented among the unique proteins of blood NPCs from HFs and BCPs. In addition, the proportion of proteins participating in ion channels and proteins binding proteins increases in the NPCs from BCP blood. However, the involvement in transport and signal transduction was greater in BCP NPCs compared to those from HFs. Gene ontology term (GO) analysis revealed that the NPC protein cargo from HF blood was enriched with proteins involved in the negative regulation of cell proliferation, and in BCP blood, proteins involved in EMT, invasion, and cell migration were observed. The combination of SPG7, ADRB1, SMCO4, PHF1, and PSMG1 NPC proteins differentiates BCPs from HFs with a sensitivity of 100% and a specificity of 80%. The obtained results indirectly indicate that, in tandem with proteins, blood cirDNA is an important part of intercellular communication, playing a regulatory and integrating role in the physiology of the body.

## 1. Introduction

Circulating DNA (cirDNA) has already proven to be a valuable tool in translational medicine [1,2,3]. However, one overlooked area of cirDNA research is its association with various proteins, despite considerable evidence that this interaction may influence the fate of DNA in the bloodstream and its molecular functions [4,5,6]. In particular, the formation of nucleoprotein complexes might allow cirDNA to be better protected from plasma nucleases [7], thereby increasing its half-life in the bloodstream. Indeed, the addition of deoxyribooligonucleotides to blood plasma leads to their rapid hydrolysis by blood enzymes [8]. There is accumulating evidence that the cell internalization of cirDNA and the initiation of biological pathways occur due to the involvement of several DNA-binding proteins, including histones [4,9]. Some nucleosome-binding proteins are able to influence immunostimulation of both free cirDNA and as part of nucleosomes [10,11,12]. Thus, the packaging shape of DNA in the bloodstream closely correlates with its molecular functions and biological effects. In 2005, a hypothesis was put forward that oncogene-containing cirDNA can transfect normal cells, leading to metastasis [13]. In 2018, it was shown that after chemotherapy, apoptotic cancer cells secrete HMGB1-containing nucleosomes that promote tumor invasion and metastasis through TLR4 and TLR9 [14]. Moreover, a number of studies have demonstrated the potential involvement of cirDNA in angiogenesis. In particular, the ability of nucleosomes to form complexes with heparin-binding angiogenic factors (FGF-1, FGF-2, VEGF, and TGFβ-1) leads to the stimulation of angiogenesis in vitro and in vivo [15]. It has also been shown that, through the activation of NF-κB/Rel-A, nucleosomes promote an increased IL-8 expression, which is involved in the early stages of angiogenesis [16]. These data suggest why hypoxic and hypervascularized areas are often revealed to be in close proximity in cancer tissues and point to the possible role of nucleosomes in the tumor progression [17].

Breast cancer is the most frequently diagnosed type of cancer among women. According to global cancer statistics, in 2020 there were 2,261,419 new cases diagnosed and 684,996 deaths worldwide [18]. Despite the effectiveness of instrumental methods for diagnosing breast cancer, there are several limitations in identifying stage one of the disease, detecting cancer in situ, differentiating benign and malignant neoplasms, and conducting screening studies of both healthy women and cancer patients after courses of treatment. To elucidate the mechanisms of breast cancer dissemination and identify promising tumor markers for liquid biopsy, intensive research is currently underway. In particular, using the Cell Death Detection ELISAplus kit (Roche Diagnostics GmbH, Mannheim, Germany), it was shown that in the blood of BCPs, the level of nucleosomes significantly increased in both node-negative and node-positive cases compared to the control group of women [19]. However, it is still unclear which proteins, other than histones, are part of the circulating DNA-protein complexes. Since proteins within circulating DNA-protein complexes in the blood may participate in the opsonization/deopsonization of circDNA and be part of the regulatory mechanism for maintaining specific DNA in circulation, a comparative analysis of proteins in nucleosomes circulating in the blood of healthy donors and cancer patients may allow us to expand fundamental knowledge of the mechanisms of tumor proliferation and identify promising proteomic cancer markers for liquid biopsy.

The aims of the study were to shed light on the differences between the proteomic cargos of histone-containing NPCs from the blood of healthy females (HFs) and breast cancer patients (BCPs) and reveal the proteins involved in carcinogenesis.

## 2. Materials and Methods

### 2.1. Blood Samples

Blood samples from HFs (*n* = 15, median age 49) were obtained from the Medical Scientific and Educational Center of the V. Zelman Institute of Medicine and Psychology, Novosibirsk State University. Only women without metabolic obesity syndrome were included in the study (in accordance with the IDF (2005) criteria) [20]. The donor group was formed on the basis of a questionnaire as well as a clinical examination. All women underwent an ultrasound examination of the breast and pelvic organs, mammography, low-dose computed tomography of the lungs, and general and biochemical blood tests. Women with reproductive system disorders, endocrine and metabolic factors, and the presence of genetic and exogenous factors were excluded from the study.

Blood samples from untreated BCPs (*n* = 20, median age 52) were obtained from the Novosibirsk Regional Clinical Oncology Dispensary. The clinicopathological parameters of BCPs are presented in Table 1.

Exclusion criteria for cancer patients from the study are as follows:

- Patients with metabolic obesity syndrome;
- Patients with the presence of hematogenous metastases at the time of surgery (M1);
- Patients who received neoadjuvant therapy.

Written informed consent was obtained from all participants and the Local Ethics Committee of the Novosibirsk State University approved the study. Blood (9 mL) was collected by venipuncture into vacutainers containing K_3_EDTA (Improvacuter, China, cat. No. 694091210), and processed as described previously [21]. The absence of hemolysis/lysis of blood cells was confirmed through the determination of LDH levels using the LDH kit as recommended by the manufacturer (Vector-Best Ltd., Novosibirsk, Russia); samples with signs of hemolysis were excluded from the study.

### 2.2. Histone-Contained NPC Isolation by Affinity Chromatography

An affine sorbent with immobilized anti-histone antibodies was synthesized as described previously [21]. Histone-containing NPCs were isolated from individual plasma samples (0.8 mL) by affinity chromatography, and the NPC samples were concentrated as described previously [21].

### 2.3. Characterization of Blood NPC Proteins

DNA from histone-containing NPCs was isolated using a BPD-100 Kit (Ltd Biosilika, Novosibirsk, Russia) according to the supplier’s protocol. Isolated DNA was concentrated by reprecipitation with trimethylamine and glycogen as described earlier [22]. The size of NPC DNA was determined with capillary electrophoresis using an Agilent 2100 Bioanalyzer^TM^ (Agilent Technologies, Waldbronn, Germany) using a “High Sensitivity DNA Kit” in the Siberian Branch of the Russian Academy of Sciences Genomics Core Facility (ICBFM SB RAS, Novosibirsk, Russia).

NPC proteins were analyzed using Laemmli gradient disk electrophoresis in 10–20% PAAG. The proteins were transferred from the gel onto a nitrocellulose membrane using a western blotting procedure and stained with colloidal silver [23].

For the NPC protein identification, the proteins were separated using SDS disk electrophoresis. The individual samples were loaded in five repeats. The gels containing the proteins were stained with Coomassie R250 (Sigma, St. Louis, MO, USA). The PAAG fragments containing the proteins under study were treated using the modified Rosenfeld method [24]. Briefly, cut PAAG fragments with proteins were washed from Coomassie R250 and SDS with a solution of 50% acetonitrile and 0.1% trifluoroacetic acid. The proteins absorbed in the gel were reduced using 45 mM DTT in 0.2 M ammonium bicarbonate at 60 °C for 30 min, followed by protein alkylation with 100 mM iodoacetamide in 0.2 M ammonium bicarbonate at room temperature for 30 min. Gel fragments were dehydrated in 100% acetonitrile. For lysine acylation, 5 µL of acetic anhydride and 40 µL of 0.1 M ammonium bicarbonate were added to the samples and incubated for 40 min at 37 °C [25]. Twenty µL of 0.2 mM trypsin (Sigma, T6567, St. Louis, MO, USA) in a mixture of 0.1 M ammonium bicarbonate and 5 µM CaCl_2_ was added to each gel piece and incubated for 30 min at room temperature. Then, 60 µL of buffer for peptide extraction was added to the gel pieces and incubated for 16–18 h at 37 °C. Peptide fragments of the proteins extracted from the gel were concentrated and desalted on C18 ZipTips microcolumns (Milipore, Darmstadt, Germany). The peptide mixture was eluted from the microcolumn on a target of the device plate with the saturated matrix solution. Mass spectra were registered at the Center of Collective Use “Mass spectrometric investigations” SB RAS on an Ultraflex III MALDI-TOF/TOF mass spectrometer (BrukerDaltonics, Bremen, Germany) in positive mode, with the range 700–3000 Da, and with 2,5-dihydroxybenzoic acid as a matrix. Proteins were identified by searching for appropriate candidates in annotated NCBI and SwissProt databases using Mascot software (Matrix Science Ltd., London, UK, www.matrixscience.com/search_form_select.html, accessed on 10 May 2023). The following parameters were used for searches: acceptable mass deviation of the charged peptide (50 ppm)—0.05 Da; acceptable number of missed cleavage sites—2; carbamidomethylation of cysteine residues was chosen as a fixed modification, and the presence of oxidized methionine residues was chosen as a variable modification; identification reliability was not lower than 95%.

### 2.4. Bioinformatics Analysis of NPC Proteins

Analysis of identified proteins was carried out using the Interpro web platform (https://www.ebi.ac.uk/interpro/ accessed on 25 May 2023), PROSITE, and Pfam databases (http://pfam.xfam.org/ accessed on 25 May 2023).

GO profiling of NPC proteins involved in the cell migration and motility, immune response, vasculature development, and cell proliferation was performed using QuickGO annotation terms (lists of obtained proteins were searched against GO terms: cell motility (GO:0048870), cell migration (GO:0016477), negative regulation of cell motility (GO:2000146), immune response (GO:0006955), negative regulation of immune response (GO:0050777), vasculature development (GO:0001944) negative regulation of vasculature development (GO:1901343), cell population proliferation (GO:0008283), and negative regulation of cell population proliferation (GO:0008285)) [26,27,28]. The involvement of NPC proteins in cancer invasion and EMT was routinely analyzed by searching the PubMed database for relevant publications for each protein. 

The sensitivity and specificity were calculated from the receiving operator characteristic curves (ROC-curves) established for discriminating patients with or without breast cancer using a commonly used method.

The search for cancer prognostic proteins in NPC proteomes was conducted using Human Protein Atlas datasets (http://www.proteinatlas.org/ accessed on 31 May 2023) for breast, renal, thyroid, pancreatic, liver, endometrial, head and neck, ovarian, stomach urothelial, cervical, lung, and colorectal cancers, as well as melanoma and glioma datasets.

## 3. Results

### 3.1. Characterization of Blood Plasma NPCs

Affinity chromatography with immobilized anti-histone antibodies was used for the isolation of NPCs circulating in the blood plasma of HFs and BCPs. 

**Figure 1:**
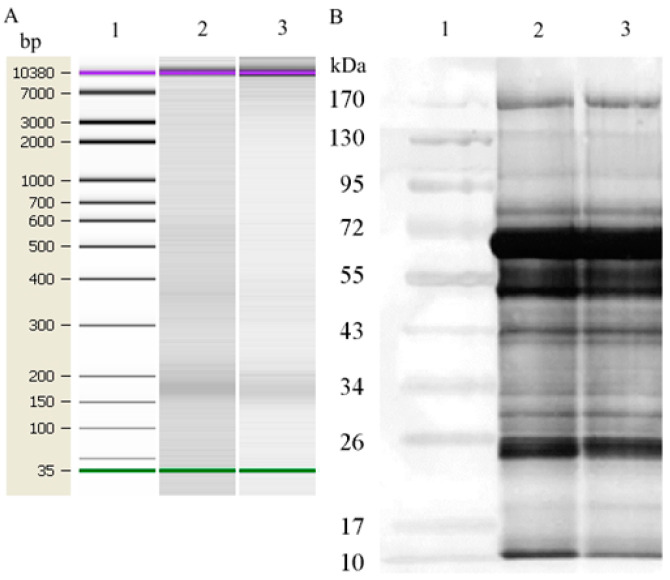
Characterization of NPCs from blood plasma of HFs and BCP. (**A**) Size of DNA extracted from NPCs. Data from the Agilent 2100 Bioanalyzer^TM^ with 35 nt and 10,380 nt DNA fragments as internal standards are shown. Legend: 1—DNA ladder; 2—DNA histone-containing NPCs from the blood of HFs; 3—DNA histone-containing NPCs from the blood of BCPs. (**B**) Molecular weights of proteins extracted from NPCs through Laemmli 10–20% gradient gel electrophoresis. The nitrocellulose membrane was stained with colloidal silver. Legend: 1—molecular weight protein PageRuler SM0671 markers (Fermentas); 2—histone-containing NPC from the blood of HFs; 3—histone-containing NPC from the blood of BCPs.

The size of the DNA in the isolated NPC samples was assessed using capillary electrophoresis following DNA isolation and concentration. Since the cirDNA concentration in the plasma of HFs was relatively low, individual samples of DNA from HF NPCs were pooled. The DNA samples from BCP NPCs were analyzed individually. It was shown that all samples mainly contained DNA-NPC fragments of ~180 bp (Figure 1A). 

A comparative electrophoretic analysis of histone-containing NPC proteins from the blood of HFs and BCPs did not show any differences in the protein spectrum. The protein motilities corresponded to those of HSA, histones, and immunoglobulins, as well as proteins with molecular masses from 11 to 170 kDa (Figure 1B).

### 3.2. Annotation of Proteins from Blood Plasma NPCs

**Figure 2:**
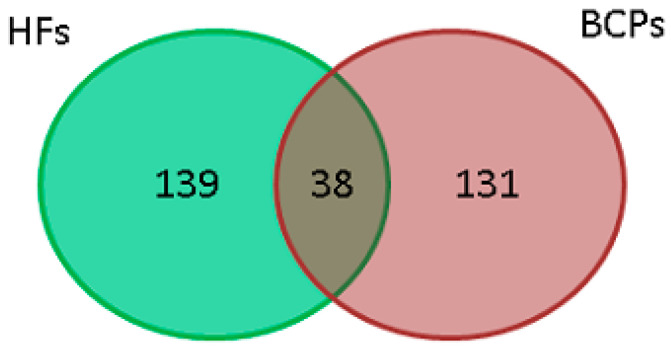
Venn–Euler diagram of proteins in NPCs from HF and BCP blood plasma.

After 1D SDS-PAGE protein separation, the whole lane was cut into 25 bands of about 2 mm each. After in-gel trypsin digestion, the peptides were extracted from each band, and then loaded to a MALDI-TOF/TOF mass spectrometer for protein identification. In total, 177 and 169 proteins (Supplementary Tables S1 and S2, Table 2) were identified with high reliability (*p* < 0.05) by MALDI-TOF mass spectrometry, in NPCs from HF and BCP blood, respectively (Figure 2). Of these, 38 proteins were common between the groups (Figure 2, Table 2). The histones H2a, H2b, and H3 were excluded from the analysis. 

To characterize proteins identified in blood-circulating NPCs, a bioinformatics analysis was performed using InterPro and InterProScan databases of versions 5.15-58 and 5.15-54,24,25, allowing us to identify the GO categories for NPC proteins from HFs (Supplementary Table S3) and BCPs (Supplementary Table S4) (isoforms not shown). 

To avoid loss of information and to fully account for the data obtained, all proteins were included in the bioinformatics analysis (even if a protein occurred in only one sample). The resulting lists of GO terms for 36 cellular components, 125 molecular functions, and 106 biological processes are provided in Supplementary Tables S5–S7. For 46 proteins, the GO terms were not determined for all three categories (cellular components, molecular function, and biological process) (Supplementary Table S8).

**Figure 3:**
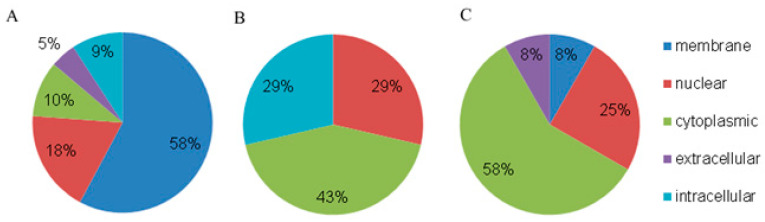
GO analysis of NPC proteins by cellular components. (**A**) Universal proteins, (**B**) unique proteins of HFs, and (**C**) unique proteins of BCPs.

The protein analysis from blood plasma NPCs of HFs and BCPs in the category of GO “Cellular components” revealed that among the universal categories, membrane proteins account for almost half of all identified proteins (terms: integral component of membrane (GO:0016021), integral component of plasma membrane (GO:0005887), membrane (GO:0016020), and postsynaptic membrane (GO:0045211)). These results may indirectly indicate the possibility of cirDNA binding to the cell surface, which is probably a prerequisite for cell internalization and an influence on its biological processes. The proportion of extracellular proteins in the composition of NPCs is less than 5%, which also indirectly indicates that already formed NPCs enter circulation (Figure 3A).

The unique proteins in the NPCs of HF blood were also categorized by the following localizations: cytoplasmic, intracellular, and nuclear proteins (Figure 3B). Most of the unique proteins of BCP blood NPCs were presented as cytoplasmic and nuclear proteins (Figure 3C).

Thus, the unique proteins of NPCs from HFs differ from the unique proteins of NPCs from BCPs by the presence of intracellular proteins and the absence of membrane and extracellular proteins.

**Figure 4:**
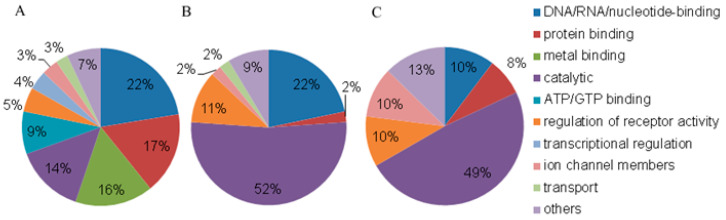
GO analysis of NPC proteins by molecular functions. (**A**) Universal proteins, (**B**) unique proteins of HFs, and (**C**) unique proteins of BCPs.

The analysis of NPC proteins from the blood plasma of HFs and BCPs in the GO category “Molecular Functions” revealed that nucleic acid/nucleotide-binding proteins were the most represented among the universal categories (terms: nucleotide binding (GO:0000166), nucleic acid binding (GO: 0003676), DNA-binding (GO:0003677), RNA-binding (GO:0003723), and sequence-specific DNA-binding (GO:0043565)), as well as protein-binding proteins (protein binding (GO: 0005515)) and metal ions (terms: iron ion-binding (GO:0005506), calcium ion-binding (GO:0005509), zinc ion-binding (GO:0008270), and metal ion-binding (GO:0046872)) (Figure 4A). 

These results indirectly confirm that a portion of the DNA-binding proteins have a “zinc finger” motif. In addition, a proportion of the NPC proteins with protein-binding and non-DNA-binding ability appear to be “passengers” in the circulating complexes in the blood. Catalytically active proteins, as well as proteins that bind nucleic acids and regulate the activity of receptors (Figure 4B,C), are the most represented among the unique proteins of blood NPCs from HFs and BCPs. In addition, the proportion of proteins participating in ion channels and protein binding proteins increases in NPCs from BCP blood (Figure 4C).

**Figure 5:**
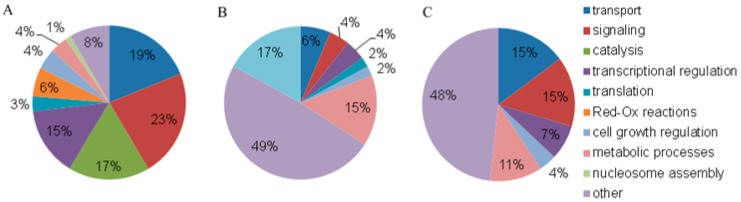
GO-analysis of NPC proteins by biological processes. (**A**) Universal proteins, (**B**) unique proteins of HFs, and (**C**) unique proteins of BCPs.

The analysis of NPC proteins from the blood of HFs and BCPs in the GO category “Biological Process” revealed proteins involved in signal transduction (terms: signal transduction (GO:0007165), signal transduction mediated by small GTPases (GO:0007264), intracellular signal transduction (GO: 0035556), G-protein coupled receptor signaling (GO:0007186), and G-protein coupled adenylate cyclase activating receptor (GO:0007189)), as well as in transport (terms: transport (GO:0006810), ion transport (GO:0006811), anion transport (GO: 0006820), chloride (GO:0006821), lipid (GO:0006869), protein (GO:0015031), and intracellular (GO:0006886) and transmembrane (GO:0055085) transport), and in transcription regulation (term GO:0006355), as well as carrying out catalytic reactions (Figure 5A).

Proteins involved in transport and signal transmission are most represented in the NPCs from cancer patients compared to those from healthy donors, while proteins involved in RNA modification and processing are absent (Figure 5B,C), which may indirectly explain the reason for the increase in the concentration of cirDNA in the blood during the development of neoplasms.

Currently, it is unknown whether a specific assembly of NPCs is carried out when cirDNA is released in the form of complexes with nucleosomes, and whether they, like exosomes, are a molecular imprint of the secreting cell, including hyperexpressed proteins as “passenger” proteins.

### 3.3. Involvement of NPC Proteins in Tumor Dissemination 

GO annotation of the NPC proteins revealed numerous proteins involved in the crucial steps of tumor dissemination such as EMT, cell motility, vasculature development, invasion, cell proliferation, and immune response (Table 3). 

It was discovered that five BCP NPC proteins (4%) are involved in EMT, while the HF NPCs contained only one EMT-related protein (0.7%), and no proteins involved in EMT were found in the universal proteins. The representation of proteins involved in cell proliferation was quite similar (31 (24%) in BCPs vs. 29 (21%) in HFs). However, only two of these proteins were involved in the negative regulation of cell proliferation in BCPs, while in HFs, nine proteins have the negative regulation of cell proliferation GO terms associated with them. Also, 14 (11%) unique NPC proteins were involved in the invasion-associated functions in BCPs, 14 (11%) in HFs, and 9 (7%) in universal proteins, respectively. Of note, none of the groups contained proteins involved in the invasion suppression. Moreover, 24 (18%) of NPC proteins unique to the BCPs were involved in cell migration, while HF NPCs contained only 16 (12%) of the cell migration-associated proteins. In terms of the vasculature development, BCP NPCs displayed two of such unique proteins, while HFs displayed one. Moreover, none of them had the inhibitory terms associated. However, one protein, mentioned as an inhibitor of the vasculature development, has been revealed from four NPC proteins, common for both groups. The BCP NPCs also contained twelve proteins (9%) that are involved in the immune response, one of which is associated with the immune suppression; HF NPCs contained five such proteins (4%), none of which are associated with the immune suppression. 

Thus, it is shown that the NPCs from HF blood are enriched with proteins involved in the negative regulation of the cell proliferation, and in BCPs they are enriched with proteins involved in EMT, invasion, and cell migration. 

### 3.4. NPC Proteins as Potential Markers for Liquid Biopsy of Breast Cancer

**Figure 6:**
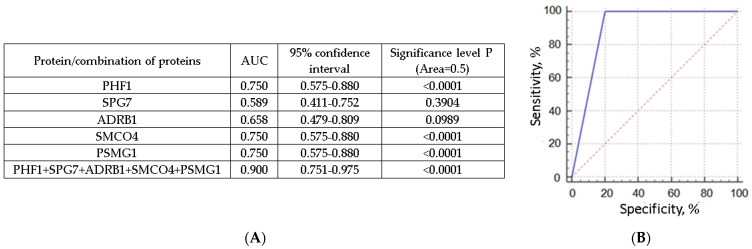
(**A**) AUC and 95% confidence interval for individual protein marker. (**B**) Approximated ROC-curve for the determination of SPG7, ADRB1, SMCO4, PHF1, and PSMG1 proteins in plasma NPCs.

The combination of SPG7, ADRB1, SMCO4, PHF1, and PSMG1 NPC proteins (Table 4) allows for the differentiation of untreated BCPs in the initial stages of disease (T1N0M0) from HFs with a sensitivity of 100% and a specificity of 80% (Figure 6), while the reduction of the diagnostic panel for the proteomic marker SMCO4 leads to a decrease in the sensitivity to 89% while maintaining the specificity of 80%. Moreover, potential breast cancer markers (PHF1, SPG7, ADRB1, SMCO4, and PSMG1) were analyzed using datasets available in the Human Protein Atlas (www.proteinatlas.org/, accessed on 31 May 2023). The PHF1 protein was shown to be overexpressed, while the SMCO4 and PSMG1 proteins had medium expression levels in breast carcinoma cells, all with low tissue specificity. There is no information on the expression levels of the ADRB1 and SPG7 proteins in breast tissues, or their diagnostic and prognostic significance in the Human Protein Atlas database.

## 4. Discussion

An increased concentration of cirDNA is a hallmark of tumor progression [29]. Currently, most studies are aimed at detecting the tumor-associated changes in cirDNA from the blood of cancer patients. These changes include mutations in genes for factors inducing cell division and tumor suppressor genes [30,31], genetic instability, which leads to the microsatellite alterations [32] and a loss of heterozygosity [33], and aberrant methylation of oncogenes and tumor suppressor genes [34,35]. Previously, the effect of blood DNases on DNA concentration was shown [7,36], but it is unknown whether the composition of proteins in NPCs that protect DNA from degradation affects the duration of DNA circulation. Moreover, it is still unknown how the composition of NPC proteins changes in cancer, and how changes in the NPC proteomic portrait affects the biological role of DNA.

In the current work, the composition of native histone-containing NPCs circulating in the blood of HFs and BCPs is described for the first time. The data from the GO-analysis of NPC proteins by cellular components indirectly indicate that already formed NPCs enter the blood (the proportion of extracellular proteins is only 5%). In addition, the unique NPC proteins in the HF blood differ from the unique NPC proteins in the BCP blood by the presence of intracellular proteins and the absence of membrane and extracellular proteins, which indirectly indicate the different mechanisms of formation of the NPC under normal and pathological conditions.

Comparative analysis of the composition of unique proteins in normal and breast cancer NPCs by their molecular functions showed a 4.7-fold increase in the proportion of proteins participating in ion channels and a 3.5-fold increase in the proportion of protein-binding proteins, as well as a 2-fold decrease in the proportion of DNA-binding proteins during the development of the cancer pathology. The data obtained may indicate a significant contribution of ion channels to DNA transport to/from the cell and the importance of “passenger” proteins that do not directly bind DNA, but are participants in circulating NPCs. Such “passenger” proteins can both serve for the targeted DNA delivery and influence the biological processes in the recipient cells.

As a result of the GO analysis of the biological functions of unique NPC proteins, it was shown that in breast cancer, the proportion of transport proteins increased by 2.5 times and the proportion of signal proteins increased by 3.8 times. Moreover, the protein cargo of the NPCs from HF blood was enriched with proteins negatively regulating cell proliferation, and in BCPs, the NPCs are enriched with proteins involved in EMT, invasion, and cell migration. The results obtained once again highlight that blood-circulating DNA is not just a means of garbage disposal during the cell lifespan, but an important part of the intercellular communication, performing a regulatory and integrating role in the physiology of the body in tandem with proteins.

The involvement of the proteins SPG7, ADRB1, SMCO4, PHF1, and PSMG1 in breast carcinogenesis is not well understood. The *SPG7* gene is implicated in the development of a genetically heterogeneous group of neurodegenerative diseases. In sporadic breast cancer samples with LOH at 16q24.3, single nucleotide polymorphisms in exon 11, intron 7, intron 10, and intron 12 were detected in the *SPG7* gene [37]. The SPG7 protein was later shown to be an important part of the mitochondrial permeability transition pore. Disorders in this mitochondrial permeability transition can lead to oxidative stress or the dysfunction of mitochondria-dependent apoptosis, and as a consequence, malignant cell degeneration [38]. Neurovascular factors are known to be involved in the development and metastasis of some malignant tumors [39,40]. It was shown that the product of adrenergic receptor gene *ADRB1* blockers have the ability to reduce the risk of many cancer, including breast cancer [41,42,43,44]. The role of PHF1 in carcinogenesis is still very much in the dark. In particular, the Polycomb group protein PHF1 is known to stimulate the H3K27-methyltransferase activity of the PRC2 complex in vitro and in vivo [45,46]. In addition to the roles in gene repression, PHF1 is also involved in the response to DNA double-strand breaks in human cells. PHF1 is rapidly recruited to double-strand break sites, promoting non-homologous end-joining processes through direct interaction with Ku70/Ku80 [47]. PHF1 was also found to stabilize p53 by promoting cell growth arrest and apoptosis, protecting p53 from MDM2-mediated ubiquitination and degradation, with PHF1 expression significantly reduced in breast cancer [48]. A model combining machine learning and explainable artificial intelligence methods was created to predict breast cancer metastasis. It was found that the reduced levels of expression of the CACTIN, TGFB3, SCUBE2, ARL4D, OR1F1, ALDH4A1, PHF1, and CROCC (*p* ≤ 0.05) genes increase the risk of metastasis in breast cancer [49]. The association between the PSMG gene family and cancer remains largely undescribed. Earlier studies have shown that PSMG1 is associated with increased susceptibility to inflammatory bowel disease, which can lead to diseases associated with colon cancer [50,51], whereas the co-expression relationship of NUP37 with PSMG1 was proposed to play a specific role in breast cancer [52]. In addition, the targeting of PSMG1 caused by miR-484 inhibition resulted in decreased cell migration and invasion in prostate cancer [53]. The role of SMCO4 in cancer progression has not yet been reported; however, the protein is shown to be a transmembrane protein involved in cellular signaling.

Promising proteomic markers of breast cancer (SPG7, ADRB1, SMCO4, PHF1, and PSMG1) from NPCs identified in this study should be further verified in larger patient groups. In the combination with tumor cirDNA sequence analysis, these tumor-associated NPC proteins may serve as potential markers for the development of multimarker approaches for early non-invasive diagnosis of malignant neoplasms.

## Figures and Tables

**Table 1:** Clinicopathological characteristics of untreated luminal BCPs (ER+/PR+/HER2−).

		N (%)
Tumor stage	T1	20 (100%)
Lymph node status	N0	20 (100%)
Distant metastasis	M0	20 (100%)
Histologic grade	II III	19 (95%) 1 (5%)
Histological type	Invasive ductal carcinoma	20 (100%)

**Table 2:** Universal NPC proteins identified in the blood of HFs and BCPs.

Uniprot ID	Protein Name	Gene Name	Score	Detected in More Than 75% of Samples
Q7Z5R6	Amyloid beta A4 precursor protein-binding family B member 1-interacting protein	APBB1IP	63	
P08588	Beta-1 adrenergic receptor	ADRB1	60	+
Q6Y288	Beta-1,3-glucosyltransferase	B3GALTL	94	
Q03060	cAMP-responsive element modulator	CREM	56	+
A2IDD5	Coiled-coil domain-containing protein 78	CCD78	70	
Q7Z7J5	Developmental pluripotency-associated protein 2	DPPA2	71	
A8MZ26	EF-hand calcium-binding domain-containing protein 9	EFCAB9	65	+
Q9BY07	Electrogenic sodium bicarbonate cotransporter 4	SLC4A5	78	
Q02108	Guanylate cyclase soluble subunit alpha-3	GUCY1A3	60	
Q00444	Homeobox protein Hox-C5	HOXC5	92	+
Q8NBZ0	INO80 complex subunit E	INO80E	89	
P14735	Insulin-degrading enzyme	IDE	77	+
O00522	Krev interaction trapped protein 1	KRIT1	70	
Q96AQ8	Mitochondrial calcium uniporter regulator 1	CCDC90A	60	
Q99558	Mitogen-activated protein kinase kinase kinase 14	MAP3K14	61	
P19105	Myosin regulatory light chain 12A	MYL12A	60	+
P24844	Myosin regulatory light polypeptide 9	MYL9	57	
P48163	NADP-dependent malic enzyme	MAOX	73	
P48745	NOV homolog	NOV	57	
Q9UQ90	Paraplegin	SPG7	61	
O75570	Peptide chain release factor 1, mitochondrial	MTRF1	70	
Q99680	Probable G-protein coupled receptor 22	GPR22	76	+
Q5JUK9	Putative G antigen family D member 1	PAGE3	56	+
A8MU76	Putative UPF0607 protein ENSP00000381418	N/A	60	
A8MX80	Putative UPF0607 protein ENSP00000383144	YM017	68	
P11233	Ras-related protein Ral-A	RALA	59	
Q8WU08	Serine/threonine-protein kinase 32A	STK32A	68	
P62314	Small nuclear ribonucleoprotein Sm D1	SNRPD1	65	
Q8NHX4	Spermatogenesis-associated protein 3	SPATA3	67	+
Q01081	Splicing factor U2AF 35 kDa subunit	U2AF1	112	+
Q13033	Striatin-3	STRN3	66	
Q7Z422	SUZ domain-containing protein 1	SZRD1	58	
Q9H2G4	Testis-specific Y-encoded-like protein 2	TSPYL2	79	
P17535	Transcription factor jun-D	JUND	68	+
Q15629	Translocating chain-associated membrane protein 1	TRAM1	73	
Q8N609	Translocating chain-associated membrane protein 1-like 1	TRAM1L1	62	+
Q9UPT9	Ubiquitin carboxyl-terminal hydrolase 22	UBP22	68	
Q16763	Ubiquitin-conjugating enzyme E2	UBE2S	63	

**Table 3:** NPC proteins from HFs’ and BCPs’ blood associated with carcinogenesis *.

Protein	EMT	Cell Proliferation	Invasion	Cell Migration	Vasculature Development	Immune Response
A0JLT2						
A6NJT0						
ACOT11						
O00159						
O00401						
O00522						
O14929						
O15392						
O43307						
O43918						
O75084						
O75190						
O75317						
O75912						
O95236						
O95260						
O95837						
O96004						
O96020						
P02795						
P06858						
P08311						
P08588						
P11233						
P14316						
P15692						
P17482						
P17483						
P17535						
P17812						
P17987						
P20592						
P23443						
P23942						
P24844						
P29371						
P29466						
P31271						
P34896						
P43487						
P46777						
P48163						
P48995						
P49137						
P49757						
P50454						
P51665						
P55010						
P60059						
P60608						
P62314						
Q01081						
Q02108						
Q03060						
Q03252						
Q05215						
Q06416						
Q13033						
Q13111						
Q13114						
Q13118						
Q13243						
Q13454						
Q14332						
Q15391						
Q15404						
Q15629						
Q15645						
Q16206						
Q3YEC7						
Q5T447						
Q5T4B2						
Q5VIR6						
Q69YI7						
Q6NXG1						
Q6PCT2						
Q6STE5						
Q75V66						
Q7LGA3						
Q7Z422						
Q7Z5R6						
Q7Z6I6						
Q7Z7J5						
Q8IV76						
Q8IX30						
Q8N1L4						
Q8NEC5						
Q8WU08						
Q92623						
Q96AQ8						
Q96HJ3						
Q99442						
Q99558						
Q99608						
Q99626						
Q99683						
Q99784						
Q99999						
Q9BT49						
Q9BY07						
Q9BYE3						
Q9H000						
Q9H239						
Q9H2G4						
Q9H6E4						
Q9H6Y7						
Q9HCP0						
Q9NS84						
Q9NYL4						
Q9NZE8						
Q9NZU5						
Q9P126						
Q9UKG4						
Q9ULL5						
Q9UPG8						
Q9UQ90						
Q9Y3C8						
Q9Y3M8						

* Proteins that negatively regulate the process are marked with hatching. Legend: 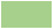 HF; 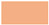 BCP; 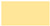 universal.

**Table 4:** Potential proteomic markers of breast cancer in the composition of circulating NPCs in blood.

Protein Name	Gene Name	Protein Description
PHD finger protein 1	PHF1	Zinc-binding protein is a component of a methyltransferase complex specific for Lys-27 of histone H3 (H3K27); it is involved in the repression of homeotic gene transcription. The protein is also recruited to double-strand breaks, and decreased levels of the protein result in sensitivity to X-rays and increased homologous recombination.
Beta-1 adrenergic receptor	ADRB1	An integral membrane protein that mediates catecholamine-induced activation of adenylate cyclase through the action of G-proteins. This receptor binds adrenaline and noradrenaline with approximately equal affinity. Mediates the activation of Ras through G(s)-α- and cAMP-mediated signaling. Also present in the early endosome.
Proteasome assembly chaperone 1	PSMG1	A cytoplasmic/nuclear chaperone protein that promotes assembly of the 20S proteasome as part of a heterodimer with PSMG2. The PSMG1-PSMG2 heterodimer binds to PSMA5 and PSMA7 proteasome subunits, promotes assembly of the α-subunits of the proteasome into a heteroheptameric α-ring, and prevents dimerization of the α-ring.
Single-pass membrane and coiled-coil domain-containing protein 4	SMCO4	The transmembrane protein. 17-β-estradiol decreases and cisplatin increases SMCO4 mRNA expression.
Paraplegin	SPG7	Transmembrane ATP-dependent zinc metalloprotease that is involved in protein folding and proteolysis.

## Data Availability

The data presented in this study are available on request from the corresponding author.

## Supplementary Materials

The following supporting information can be downloaded at: https://www.mdpi.com/article/10.3390/jpm13121691/s1. Table S1: Unique NPC proteins identified in the plasma of HF blood; Table S2: Unique NPC proteins identified in the plasma of BCP blood; Table S3: HF NPC proteins and their predicted InterPro classification and GO terms; Table S4: BCP NPC proteins and their predicted InterPro classification and GO categories; Table S5: Summary list of Cellular Components GO terms based on the functional enrichment analysis results for NPC proteins in the HF and BCP blood; Table S6: Summary list of Molecular Function GO terms based on the functional enrichment analysis results for NPC proteins in the HF and BCP blood; Table S7: Summary list of Biological Processes GO terms based on the functional enrichment analysis results for NPC proteins in the HF and BCP blood; Table S8: Proteins for which InterPro and GO database categories have not been attributed.
