# Supplementary material for: Blood Plasma Circulating DNA-Protein Complexes: Involvement in Carcinogenesis and Prospects for Liquid Biopsy of Breast Cancer
Source: J Pers Med. 2023 Dec 5;13(12):1691. doi: 10.3390/jpm13121691 (PMC10744380; doi:10.3390/jpm13121691)
Supplement: Supplementary file 1 [file jpm-13-01691-s001.zip › jpm-2686504-supplementary.pdf]

SUPPL TABLE S1 Unique NPC proteins identified in the plasma of HF blood

UniprotID	Protein Name	Gene Name	Score
P51665	26S proteasome non-ATPase regulatory subunit 7	PSMD7	66
Q92665	28S ribosomal protein S31, mitochondrial	MRPS31	70
Q9HD33	39S ribosomal protein L47, mitochondrial	MRPL47	56
Q6H8Q1	Actin-binding LIM protein 2	ABLIM2	61
Q8WXI4	Acyl-coenzyme A thioesterase 11	ACOT11	62
P40123	Adenylyl cyclase-associated protein 2	CAP2	66
P24298	Alanine aminotransferase 1	GPT	61
P18825	Alpha-2C adrenergic receptor	ADRA2C	57
Q96LR9	Apolipoprotein L domain-containing protein 1	APOLD1	62
O43918	Autoimmune regulator	AIRE	61
P08311	Cathepsin G	CTSG	68
P29973	cGMP-gated cation channel alpha-1	CNGA1	60
Q8TDX6	Chondroitin sulfate N-acetylgalactosaminyltransferase 1	CSGALNACT1	60
Q13111	Chromatin assembly factor 1 subunit A	CHAF1A	95
Q92187	CMP-N-acetylneuraminate-poly-alpha-2,8-sialyltransferase	ST8SIA4	78
Q96HJ3	Coiled-coil domain-containing protein 34	CCDC34	57
A6NFT4	Coiled-coil domain-containing protein 42B	CCDC42B	63
Q86UT8	Coiled-coil domain-containing protein 84	CCDC84	58
P08174	Complement decay-accelerating factor	CD55	75
P36980	Complement factor H-related protein 2	CFHR2	72
Q9BR76	Coronin-1B	COR1B	93
P17812	CTP synthase 1	CTPS1	64
Q9P126	C-type lectin domain family 1 member B	CLEC1B	66
Q6NT55	Cytochrome P450 4F22	CYP4F22	59
P25205	DNA replication licensing factor MCM3	MCM3	61
O75190	DnaJ homolog subfamily B member 6	DNAJB6	96
O60941	Dystrobrevin beta	DTNB	65
Q8N7E2	E3 ubiquitin-protein ligase ZNF645	ZN645	58
O75354	Ectonucleoside triphosphate diphosphohydrolase 6	ENTPD6	61
Q6NXG1	Epithelial splicing regulatory protein 1	ESRP1	79
P55010	Eukaryotic translation initiation factor 5	EIF5	70

P14324	Farnesyl pyrophosphate synthase	FPPS	57
Q6PCT2	F-box/LRR-repeat protein 19	FBXL19	57
Q5T3I0	G patch domain-containing protein 4	GPATCH4	57
O96020	G1/S-specific cyclin-E2	CCNE2	82
Q99999	Galactosylceramide sulfotransferase	GAL3ST1	59
Q92990	Glomulin	GLMN	66
P06744	Glucose-6-phosphate isomerase	GPI	67
P23415	Glycine receptor subunit alpha-1	GLRA1	62
Q7LGA3	Heparan sulfate 2-O-sulfotransferase 1	HS2ST1	72
Q8WW32	High mobility group protein B4	HMGB4	60
O14929	Histone acetyltransferase type B catalytic subunit	HAT1	56
Q9BTM1	Histone H2A.J	H2AFJ	75
P31271	Homeobox protein Hox-A13	HOXA13	67
P17483	Homeobox protein Hox-B4	HOXB4	58
P31273	Homeobox protein Hox-C8	HOXC8	65
Q92819	Hyaluronan synthase 2	HAS2	74
Q9NSI5	Immunoglobulin superfamily member 5	IGSF5	66
Q9NV88	Integrator complex subunit 9	INTS9	80
P20592	Interferon-induced GTP-binding protein Mx2	MX2	68
Q8IXL9	IQ domain-containing protein F2	IQCF2	56
O95198	Kelch-like protein 2	KLHL2	58
Q8N4N8	Kinesin-like protein KIF2B	KIF2B	60
Q13118	Krueppel-like factor 10	KLF10	68
Q03252	Lamin-B2	LMNB2	62
Q96BZ8	Leukocyte receptor cluster member 1	LENG1	68
P49137	MAP kinase-activated protein kinase 2	MAPKAPK2	86
A6NI15	Mesogenin-1	MSGN1	58
P02795	Metallothionein-2	MT2A	60
Q2M296	Methenyltetrahydrofolate synthase domain-containing protein	MTHFSD	57
Q9BVV7	Mitochondrial import inner membrane translocase subunit Tim21	TIMM21	66
Q99683	Mitogen-activated protein kinase kinase kinase 5	MAP3K5	71
Q8NB16	Mixed lineage kinase domain-like protein	MLKL	68
Q9HD90	Neurogenic differentiation factor 4	NEUROD4	72

Q9Y639	Neuroplastin	NPTN	57
Q99784	Noelin	OLFM1	64
Q96PB7	Noelin-3	OLFM3	77
Q8NGW1	Olfactory receptor 6B3	OR6B3	80
P30559	Oxytocin receptor	OXTR	73
Q15391	P2Y purinoceptor 14	P2RY14	56
Q9BRP8	Partner of Y14 and mago	WIBG	65
Q8IV76	PAS domain-containing protein 1	PASD1	66
Q9NYL4	Peptidyl-prolyl cis-trans isomerase FKBP11	FKBP11	87
Q9BY49	Peroxisomal trans-2-enoyl-CoA reductase	PECR	63
Q9BUL5	PHD finger protein 23	PHF23	65
Q8N4E4	Phosducin-like protein 2	PDCL2	63
Q6NWY9	Pre-mRNA-processing factor 40 homolog B	PRPF40B	63
Q9H000	Probable E3 ubiquitin-protein ligase makorin-2	MKRN2	68
Q5JPH6	Probable glutamate--tRNA ligase, mitochondrial	EARS2	59
A2RTX5	Probable threonine-tRNA ligase 2, cytoplasmic	TARSL2	57
Q9ULL5	Proline-rich protein 12	PRR12	58
Q2TB18	Protein asteroid homolog 1	ASTE1	68
Q9UKY7	Protein CDV3 homolog	CDV3	61
Q13394	Protein mab-21-like 1	MAB21L1	57
O15151	Protein Mdm4	MDM4	58
P00734	Prothrombin	F2	59
Q8N1L4	Putative inactive cytochrome P450 family member 4Z2	CYP4Z2P	59
Q5I0G3	Putative malate dehydrogenase 1B	MDH1B	86
Q9Y383	Putative RNA-binding protein Luc7-like 2	LUC7L2	61
Q5EBN2	Putative tripartite motif-containing protein 61	TRIM61	70
Q96NF6	Putative uncharacterized protein C8orf49	C8orf49	80
A8MUU9	Putative uncharacterized protein ENSP00000383309	YV023	66
Q9H974	Queuine tRNA-ribosyltransferase subunit QTRTD1	QTRTD1	62
Q3YEC7	Rab-like protein 6	RABL6	74
Q09MP3	RAD51-associated protein 2	RAD51AP2	62
Q86UC2	Radial spoke head protein 3 homolog	RSPH3	68
Q8IV61	Ras guanyl-releasing protein 3	RASGRP3	69

P20340	Ras-related protein Rab-6A	RAB6A	58
P11234	Ras-related protein Ral-B	RALB	62
Q7Z6I6	Rho GTPase-activating protein 30	ARHGAP30	82
Q5TG30	Rho GTPase-activating protein 40	ARHGAP40	70
O43307	Rho guanine nucleotide exchange factor 9	ARHGEF9	57
P23443	Ribosomal protein S6 kinase beta-1	RPS6KB1	69
Q96CM3	RNA pseudouridylate synthase domain-containing protein 4	RPUSD4	58
Q9Y324	rRNA-processing protein FCF1 homolog	FCF1	64
Q2I0M5	R-spondin-4	RSPO4	67
Q92599	Septin-8	SEPT8	64
P34896	Serine hydroxymethyltransferase, cytosolic	SHMT1	78
Q13243	Serine/arginine-rich splicing factor 5	SRSF5	76
Q86UX6	Serine/threonine-protein kinase 32C	STK32C	60
P50454	Serpin H1	SERPINH1	61
Q9BZQ2	SHC SH2 domain-binding protein 1-like protein	SHCBP1L	61
Q8IX30	Signal peptide, CUB and EGF-like domain-containing protein 3	SCUBE3	61
Q9NR83	SLC2A4 regulator	SLC2A4RG	75
Q9NYB5	Solute carrier organic anion transporter family member 1C1	SLCO1C1	80
Q86UG4	Solute carrier organic anion transporter family member 6A1	SLCO6A1	62
Q8WXA9	Splicing regulatory glutamine/lysine-rich protein 1	SREK1	78
Q9BQG1	Synaptotagmin-3	SYT3	75
Q15573	TATA box-binding protein-associated factor RNA polymerase I subunit A	TAF1A	58
P17987	T-complex protein 1 subunit alpha	TCP1	81
Q13114	TNF receptor-associated factor 3	TRAF3	66
Q56UQ5	TPT1-like protein	TPT1L	68
Q16514	Transcription initiation factor TFIID subunit 12	TAF12	68
Q00577	Transcriptional activator protein Pur-alpha	PURA	67
Q99442	Translocation protein SEC62	SEC62	72
Q7Z5M5	Transmembrane channel-like protein 3	TMC3_	83
Q9NXH9	tRNA (guanine(26)-N(2))-dimethyltransferase	TRMT1	58
Q16560	U11/U12 small nuclear ribonucleoprotein 35 kDa protein	SNRNP35	65
Q15695	U2 small nuclear ribonucleoprotein auxiliary factor 35 kDa subunit-related protein 1	ZRSR1	80

C9J2P7	Ubiquitin carboxyl-terminal hydrolase 17-like protein 15	USP17L15	63
Q9Y3C8	Ubiquitin-fold modifier-conjugating enzyme 1	UFC1	68
O75310	UDP-glucuronosyltransferase 2B11	UGT2B11	56
Q8IXR9	Uncharacterized protein C12orf56	C12orf56	56
Q8NEA5	Uncharacterized protein C19orf18	C19orf18	59
Q5T8R8	Uncharacterized protein C9orf66	C9orf66	60
Q5VIR6	Vacuolar protein sorting-associated protein 53 homolog	VPS53	62
O95670	V-type proton ATPase subunit G	ATP6V1G2	58
Q96JC4	Zinc finger protein 479	ZNF479	59

SUPPL TABLE S2 Unique NPC proteins identified in the plasma of BCP blood

UniprotID	Protein Name	Gene Name	Score
Q9NZE8	39S ribosomal protein L35, mitochondrial	MRPL35	70
P32754	4-hydroxyphenylpyruvate dioxygenase	HPD	58
P46777	60S ribosomal protein L5	RPL5	60
P23526	Adenosylhomocysteinase	AHCY	58
Q969X2	Alpha-N-acetylgalactosaminide alpha-2,6-sialyltransferase 6	ST6GALNAC6	62
P48751	Anion exchange protein 3	SLC4A3	81
Q75V66	Anoctamin-5	ANO5	77
O95236	Apolipoprotein L3	APOL3	56
Q86W34	Archaemetzincin-2	AMZ2	59
Q8TF01	Arginine/serine-rich protein PNISR	PNISR	68
O95260	Arginyl-tRNA-protein transferase 1	ATE1	64
O15392	Baculoviral IAP repeat-containing protein 5	BIRC5	56
Q9NS84	Carbohydrate sulfotransferase 7	CHST7	70
Q9HCP0	Casein kinase I isoform gamma-1	CSNK1G1	93
Q5EG05	Caspase recruitment domain-containing protein 16	CARD16	204
P29466	Caspase-1	CASP1	64
Q8NEC5	Cation channel sperm-associated protein 1	CATSPER1	61
Q9H6E4	Coiled-coil domain-containing protein 134	CCDC134	57
Q03591	Complement factor H-related protein 1	CFHR1	60
P61201	COP9 signalosome complex subunit 2	COPS2	76
P21728	D(1A) dopamine receptor	DRD1	63
O75912	Diacylglycerol kinase iota	DGKI	60
O95886	Disks large-associated protein 3	DLGAP3	57
Q9H1X3	DnaJ homolog subfamily C member 25	DNAJC25	117
Q5T447	E3 ubiquitin-protein ligase HECTD3	HECTD3	63
Q9H6Y7	E3 ubiquitin-protein ligase RNF167	RNF167	62
Q6AZZ1	E3 ubiquitin-protein ligase TRIM68	TRIM68	68
Q05215	Early growth response protein 4	EGR4	67
Q16206	Ecto-NOX disulfide-thiol exchanger 2	ENOX2	58
A0FGR8	Extended synaptotagmin-2	ESYT2	60
Q14332	Frizzled-2	FZD2	72

O75084	Frizzled-7	FZD7	94
Q86XJ1	GAS2-like protein 3	GAS2L3	82
P48167	Glycine receptor subunit beta	GLRB	85
Q08379	Golgin subfamily A member 2	GOLGA2	63
Q99578	GTP-binding protein Rit2	RIT2	64
O95837	Guanine nucleotide-binding protein subunit alpha-14	GNA14	91
O96004	Heart- and neural crest derivatives-expressed protein 1	HAND1	61
Q92598	Heat shock protein 105 kDa	HSPH1	69
P60608	HERV-F(c)2_7q36.2 provirus ancestral Env polyprotein	EFC2_HUMAN	58
Q99626	Homeobox protein CDX-2	CDX2	65
P17482	Homeobox protein Hox-B9	HOXB9	62
A6NJT0	Homeobox protein unc-4 homolog	UNCX	58
P14316	Interferon regulatory factor 2	IRF2	57
Q8IYV9	Izumo sperm-egg fusion protein 1	IZUMO1	60
Q5VZ72	Izumo sperm-egg fusion protein 3	IZUMO3	56
Q7Z3Y9	Keratin, type I cytoskeletal 26	K1C26	57
Q96EK5	KIF1-binding protein	KIAA1279	75
Q9BYE3	Late cornified envelope protein 3D	LCE3D_HUMAN	59
Q9NZU5	LIM and cysteine-rich domains protein 1	LMCD1	66
P06858	Lipoprotein lipase	LPL	63
Q68DH5	LMBR1 domain-containing protein 2	LMBRD2	57
Q9H239	Matrix metalloproteinase-28	MMP28	74
A0JLT2	Mediator of RNA polymerase II transcription subunit 19	MED19	70
Q9P0P8	Mitochondrial transcription rescue factor 1	MTRES1	105
Q9P2K5	Myelin expression factor 2	MYEF2	64
P05976	Myosin light chain 1/3, skeletal muscle isoform	MYL1	60
O14950	Myosin regulatory light chain 12B	MYL12B	57
E9PAV3	Nascent polypeptide-associated complex subunit alpha	NACA	57
Q99608	Necdin	NDN	78
O00401	Neural Wiskott-Aldrich syndrome protein	WASL	60
Q13562	Neurogenic differentiation factor 1	NEUROD1	66
P29371	Neuromedin-K receptor	TACR3	67
Q69YI7	Nuclear apoptosis-inducing factor 1	NAIF1	62

Q9Y3N9	Olfactory receptor 2W1	OR2W1	58
Q15645	Pachytene checkpoint protein 2 homolog	TRIP13	93
Q8TE04	Pantothenate kinase 1	PANK1	69
Q96NR3	Patched domain-containing protein 1	PTCHD1	65
F5H284	Peptidyl-prolyl cis-trans isomerase A-like 4D	PPIAL4D	60
P23942	Peripherin-2	PRPH2	69
O43189	PHD finger protein 1	PHF1	67
Q9P215	Pogo transposable element with KRAB domain	POGK	69
Q9NZM6	Polycystic kidney disease 2-like 2 protein	PKD2L2	69
Q5SY16	Polynucleotide 5'-hydroxyl-kinase NOL9	NOL9	59
Q96KK3	Potassium voltage-gated channel subfamily S member 1	KCNS1	66
Q6PIU1	Potassium voltage-gated channel subfamily V member 1	KCNV1	60
P61758	Prefoldin subunit 3	VBP1	90
Q5T4B2	Probable inactive glycosyltransferase 25 family member 3	CERCAM	96
O95456	Proteasome assembly chaperone 1	PSMG1	66
O15234	Protein CASC3	CASC3	60
P49757	Protein numb homolog	NUMB	66
B4DS77	Protein shisa-9	SHISA9	68
P60059	Protein transport protein Sec61 subunit gamma	SEC61G	61
Q06416	Putative POU domain, class 5, transcription factor 1B	POU5F1B	90
Q96IC2	Putative RNA exonuclease NEF-sp	44M2.3	60
Q9Y6Q9	Putative uncharacterized protein ENSP00000380701	YQ045_HUMAN	61
A8MV72	Putative UPF0607 protein ENSP00000382826	N/A	82
Q92670	Putative zinc finger protein 75C	ZNF75CP	57
P43487	Ran-specific GTPase-activating protein	RANBP1	56
Q15404	Ras suppressor protein 1	RSU1	63
Q5HYW3	Retrotransposon gag domain-containing protein 4	RGAG4	62
Q9UJK0	Ribosome biogenesis protein TSR3 homolog	TSR3	72
A6NCQ9	RING finger protein 222	RNF222	93
Q5JTH9	RRP12-like protein	RRP12	70
P48995	Short transient receptor potential channel 1	TRPC1	62
Q8N7X8	SIGLEC family-like protein 1	SIGLECL1	65
Q9UKG4	Solute carrier family 13 member 4	SLC13A4	61

Q9BQ15	SOSS complex subunit B1	NABP2	56
Q9HB58	Sp110 nuclear body protein	SP110	65
Q8NB90	Spermatogenesis-associated protein 5	SPATA5	58
Q9Y3M8	StAR-related lipid transfer protein 13	STARD13	57
Q6STE5	SWI/SNF-related matrix-associated actin-dependent regulator of chromatin subfamily D member 3	SMARCD3	66
Q6XYQ8	Synaptotagmin-10	SYT10	60
Q9BXF9	Tektin-3	TEKT3	117
Q49AM3	Tetratricopeptide repeat protein 31	TTC31	88
Q6PGP7	Tetratricopeptide repeat protein 37	TTC37	62
Q92623	Tetratricopeptide repeat protein 9A	TTC9	61
Q9BT49	THAP domain-containing protein 7	THAP7	68
P05412	Transcription factor AP-1	JUN	73
Q9Y5Q3	Transcription factor MafB	MAFB	61
Q9UM00	Transmembrane and coiled-coil domain-containing protein 1	TMCO1	66
Q96AN5	Transmembrane protein 143	TMEM143	74
Q9H813	Transmembrane protein 206	TMEM206	60
Q9Y2B1	Transmembrane protein 5	TMEM5	62
Q7Z4G4	tRNA (guanine(10)-N2)-methyltransferase homolog	TRMT11	58
Q9UJT0	Tubulin epsilon chain	TUBE1	65
Q13454	Tumor suppressor candidate 3	TUSC3	67
O75317	Ubiquitin carboxyl-terminal hydrolase 12	USP12	68
Q8WUN7	Ubiquitin domain-containing protein 2	UBTD	58
Q96C57	Uncharacterized protein C12orf43	C12orf43	68
Q6ZW13	Uncharacterized protein C16orf86	C16orf86	93
O00159	Unconventional myosin-Ic	MYO1C	65
Q9NRQ5	UPF0443 protein C11orf75	C11orf75	56
P15692	Vascular endothelial growth factor A	VEGFA	61
O60504	Vinexin	SORBS3	66
Q52LC2	V-type proton ATPase subunit S1-like protein	ATP6AP1L	60
Q8TAF7	Zinc finger protein 461	ZNF461	58
Q96N20	Zinc finger protein 75A	ZNF75A	68
P51815	Zinc finger protein 75D	ZNF75D	57
Q9UPG8	Zinc finger protein PLAGL2	PLAGL2	61

SUPPL TABLE S3. HF NPC proteins and their predicted InterPro classification and GO terms

Protein	InterPro classification	GO (Biological Process)	GO (Molecular Function)	GO (Cellular Component)
A2IDD5.1	IPR029329	None predicted	None predicted	None predicted
A2RTX5.1	IPR002320; IPR012675; IPR012676; IPR004095; IPR018163; IPR012947; IPR006195; IPR002314; IPR033728; IPR004154	GO:0006418 tRNA aminoacylation for protein translation; GO:0006435 threonyl-tRNA aminoacylation; GO:0043039 tRNA aminoacylation	GO:0000166 nucleotide binding; GO:0004812 aminoacyl-tRNA ligase activity; GO:0004829 threonine-tRNA ligase activity; GO:0005524 ATP binding; GO:0016876 ligase activity, forming aminoacyl-tRNA and related	GO:0005737 cytoplasm
A6NFT4.3	IPR025252	None predicted	None predicted	None predicted
A6NI15.1	IPR011598	None predicted	GO:0046983 protein dimerization activity	None predicted
A8MU76.2		None predicted	None predicted	None predicted
A8MUU9.3		None predicted	None predicted	None predicted
A8MX80.2		None predicted	None predicted	None predicted
A8MZ26.2	IPR011992; IPR002048	None predicted	GO:0005509 calcium ion binding	None predicted
C9J2P7.1	IPR028889; IPR001394; IPR006861; IPR018200	GO:0006511 ubiquitin-dependent protein catabolic process; GO:0016579 protein deubiquitination	GO:0036459 thiol-dependent ubiquitinyl hydrolase activity	None predicted
O00522.2	IPR032022; IPR020683; IPR019749; IPR000299; IPR014352; IPR019748; IPR002110	None predicted	GO:0005515 protein binding	GO:0005856 cytoskeleton

O14929.1	IPR017380; IPR019467; IPR016181	GO:0006348 chromatin silencing at telomere; GO:0016568 chromatin modification; GO:0016573 histone acetylation	GO:0004402 histone acetyltransferase activity	GO:0005634 nucleus
O15151.2	IPR016495; IPR015458; IPR003121; IPR001876; IPR013083; IPR001841	GO:0043066 negative regulation of apoptotic process; GO:0071157 negative regulation of cell cycle arrest	GO:0005515 protein binding; GO:0008270 zinc ion binding	GO:0005634 nucleus
O43307.3	IPR001452; IPR000219; IPR011993; IPR001849	GO:0035023 regulation of Rho protein signal transduction	GO:0005089 Rho guanyl-nucleotide exchange factor activity; GO:0005515 protein binding	None predicted
O43918.1	IPR008087; IPR004865; IPR010919; IPR000770; IPR013083; IPR011011; IPR001965; IPR019787; IPR019786	GO:0006959 humoral immune response	GO:0003677 DNA binding; GO:0005515 protein binding; GO:0008270 zinc ion binding; GO:0045182 translation regulator activity	GO:0005634 nucleus; GO:0005737 cytoplasm
O60941.1	IPR017432; IPR015153; IPR011992; IPR015154; IPR000433	None predicted	GO:0008270 zinc ion binding	None predicted
O75190.2	IPR001623; IPR018253	None predicted	None predicted	None predicted
O75310.1	IPR002213	GO:0008152 metabolic process	GO:0016758 transferase activity, transferring hexosyl groups	None predicted
O75354.3	IPR000407	None predicted	GO:0016787 hydrolase activity	None predicted
O75570.2	IPR005139; IPR014720; IPR000352	GO:0006415 translational termination	GO:0003747 translation release factor activity; GO:0016149 translation release factor activity,	GO:0005737 cytoplasm

			codon specific	
O95198.2	IPR017096; IPR011333; IPR000210; IPR011705; IPR015916; IPR006652	None predicted	GO:0005515 protein binding	None predicted
O95670.1	IPR005124	GO:0015992 proton transport	GO:0016820 hydrolase activity, acting on acid anhydrides,	GO:0016471 vacuolar proton-transporting V-type ATPase complex
O96020.1	IPR013763; IPR006671; IPR004367	None predicted	None predicted	GO:0005634 nucleus
P00734.2	IPR001314; IPR003966; IPR000294; IPR017857; IPR013806; IPR000001; IPR018992; IPR009003; IPR001254; IPR018056; IPR018114; IPR033116	GO:0006508 proteolysis; GO:0007596 blood coagulation	GO:0004252 serine-type endopeptidase activity; GO:0005509 calcium ion binding	GO:0005576 extracellular region
P02795.1	IPR003019; IPR000006; IPR017854; IPR023587; IPR018064	None predicted	GO:0046872 metal ion binding	None predicted
P06744.4	IPR001672; IPR023096; IPR018189	GO:0006094 gluconeogenesis; GO:0006096 glycolytic process	GO:0004347 glucose-6-phosphate isomerase activity	None predicted
P08174.4	IPR000436	None predicted	None predicted	None predicted
P08311.2	IPR001314; IPR009003; IPR001254; IPR018114; IPR033116	GO:0006508 proteolysis	GO:0004252 serine-type endopeptidase activity	None predicted

P08588.2	IPR000276; IPR002233; IPR000507; IPR017452	GO:0007186 G-protein coupled receptor signaling pathway; GO:0007189 adenylate cyclase-activating G-protein coupled receptor; GO:0045823 positive regulation of heart contraction	GO:0004930 G-protein coupled receptor activity; GO:0004935 adrenergic receptor activity; GO:0004940 beta1-adrenergic receptor activity	GO:0016021 integral component of membrane
P11233.1	IPR001806; IPR020849; IPR027417; IPR005225	GO:0007165 signal transduction; GO:0007264 small GTPase mediated signal transduction	GO:0005525 GTP binding	GO:0016020 membrane
P11234.1	IPR001806; IPR020849; IPR027417; IPR005225	GO:0007165 signal transduction; GO:0007264 small GTPase mediated signal transduction	GO:0005525 GTP binding	GO:0016020 membrane
P14324.4	IPR000092; IPR008949	GO:0008299 isoprenoid biosynthetic process	None predicted	None predicted
P14735.4	IPR011249; IPR011237; IPR011765; IPR007863; IPR032632; IPR001431	GO:0006508 proteolysis	GO:0003824 catalytic activity; GO:0004222 metalloendopeptidase activity; GO:0046872 metal ion binding	None predicted
P17483.2	IPR017995; IPR009057; IPR001356; IPR020479; IPR001827; IPR017970	GO:0006355 regulation of transcription, DNA-templated	GO:0003677 DNA binding; GO:0003700 transcription factor activity, sequence-specific DNA; GO:0043565 sequence-specific DNA binding	GO:0005634 nucleus
P17535.3	IPR002112; IPR005643; IPR008917; IPR004827	GO:0006355 regulation of transcription, DNA-templated	GO:0003677 DNA binding; GO:0003700 transcription factor activity, sequence-specific DNA; GO:0043565 sequence-specific DNA binding	None predicted
P17812.2	IPR004468; IPR027417;	GO:0006221 pyrimidine	GO:0003883 CTP synthase activity	None

	IPR017456; IPR029062; IPR017926	nucleotide biosynthetic process		predicted
P17987.1	IPR002423; IPR017998; IPR012715; IPR027413; IPR027410; IPR027409; IPR002194	GO:0006457 protein folding	GO:0005524 ATP binding; GO:0051082 unfolded protein binding	None predicted
P18825.2	IPR000276; IPR002233; IPR000735; IPR017452	GO:0006940 regulation of smooth muscle contraction; GO:0007186 G-protein coupled receptor signaling pathway; GO:0019229 regulation of vasoconstriction; GO:0030168 platelet activation	GO:0004930 G-protein coupled receptor activity; GO:0004935 adrenergic receptor activity; GO:0004938 alpha2-adrenergic receptor activity	GO:0016021 integral component of membrane
P19105.2	IPR011992; IPR002048; IPR015070; IPR018247	None predicted	GO:0005509 calcium ion binding	None predicted
P20340.3	IPR001806; IPR027417; IPR005225	GO:0007264 small GTPase mediated signal transduction	GO:0005525 GTP binding	None predicted
P20592.1	IPR022812; IPR001401; IPR027417; IPR030381; IPR000375; IPR020850; IPR003130; IPR019762	None predicted	GO:0003924 GTPase activity; GO:0005525 GTP binding	None predicted
P23415.2	IPR006201; IPR006028; IPR008127; IPR008128; IPR006202; IPR006029; IPR018000	GO:0006810 transport; GO:0006811 ion transport; GO:0006821 chloride transport	GO:0005230 extracellular ligand-gated ion channel activity; GO:0016594 glycine binding; GO:0016934 extracellular-glycine-gated chloride channel activity; GO:0022824 transmitter-gated ion channel activity	GO:0005887 integral component of plasma membrane; GO:0016020 membrane; GO:0016021

				integral component of membrane; GO:0045211 postsynaptic membrane
P23443.2	IPR016238; IPR011009; IPR000719; IPR000961; IPR017892; IPR017441; IPR008271	GO:0006468 protein phosphorylation; GO:0007165 signal transduction	GO:0004672 protein kinase activity; GO:0004674 protein serine/threonine kinase activity; GO:0005524 ATP binding	None predicted
P24298.3	IPR015424; IPR004839; IPR015421; IPR015422	GO:0009058 biosynthetic process	GO:0003824 catalytic activity; GO:0030170 pyridoxal phosphate binding	None predicted
P24844.4	IPR011992; IPR002048; IPR018247	None predicted	GO:0005509 calcium ion binding	None predicted
P25205.3	IPR031327; IPR008046; IPR012340; IPR027925; IPR027417; IPR001208; IPR003593; IPR018525	GO:0006260 DNA replication; GO:0006270 DNA replication initiation	GO:0003677 DNA binding; GO:0003678 DNA helicase activity; GO:0005524 ATP binding	GO:0005634 nucleus; GO:0042555 MCM complex
P29973.3	IPR005821; IPR014710; IPR018490; IPR000595; IPR032406; IPR018488	GO:0006811 ion transport; GO:0055085 transmembrane transport	GO:0005216 ion channel activity	GO:0016020 membrane
P30559.2	IPR000276; IPR001817; IPR002062; IPR017452	GO:0007186 G-protein coupled receptor signaling pathway	GO:0004930 G-protein coupled receptor activity; GO:0004990 oxytocin receptor activity; GO:0005000 vasopressin receptor activity	GO:0016021 integral component of membrane
P31271.3	IPR022067; IPR009057; IPR001356; IPR017970	GO:0006355 regulation of transcription, DNA-templated	GO:0003677 DNA binding; GO:0043565 sequence-specific DNA binding	None predicted

P31273.2	IPR009057; IPR001356; IPR020479; IPR001827; IPR000047; IPR017970	GO:0006355 regulation of transcription, DNA-templated	GO:0003677 DNA binding; GO:0003700 transcription factor activity, sequence-specific DNA; GO:0043565 sequence-specific DNA binding	None predicted
P34896.1	IPR001085; IPR015424; IPR015421; IPR015422; IPR019798	GO:0006544 glycine metabolic process; GO:0006563 L-serine metabolic process	GO:0003824 catalytic activity; GO:0004372 glycine hydroxymethyltransferase activity; GO:0016740 transferase activity; GO:0030170 pyridoxal phosphate binding	None predicted
P36980.1	IPR000436	None predicted	None predicted	None predicted
P40123.1	IPR013992; IPR017901; IPR016098; IPR013912; IPR006599; IPR018106; IPR028417	GO:0000902 cell morphogenesis; GO:0007010 cytoskeleton organization	GO:0003779 actin binding	None predicted
P48163.1	IPR001891; IPR012301; IPR016040; IPR012302; IPR015884	GO:0006108 malate metabolic process; GO:0055114 oxidation-reduction process	GO:0004470 malic enzyme activity; GO:0004471 malate dehydrogenase (decarboxylating) (NAD+) activity; GO:0051287 NAD binding	None predicted
P48745.1	IPR012395; IPR009030; IPR000867; IPR001007; IPR006208; IPR006207; IPR000884; IPR017891	GO:0001558 regulation of cell growth	GO:0005515 protein binding; GO:0005520 insulin-like growth factor binding	GO:0005576 extracellular region
P49137.1	IPR011009; IPR000719; IPR027442; IPR017441; IPR008271	GO:0006468 protein phosphorylation	GO:0004672 protein kinase activity; GO:0005524 ATP binding	None predicted
P50454.2	IPR023796; IPR023795	None predicted	None predicted	None predicted
P51665.2	IPR000555; IPR024969	None predicted	GO:0005515 protein binding	None predicted

P55010.2	IPR016189; IPR002735; IPR016190; IPR016024; IPR003307; IPR016021	GO:0006413 translational initiation	GO:0003743 translation initiation factor activity; GO:0005488 binding; GO:0005515 protein binding	None predicted
P62314.1	IPR010920; IPR001163	None predicted	None predicted	None predicted
Q00444.1	IPR017995; IPR009057; IPR001356; IPR020479; IPR001827; IPR017970	GO:0006355 regulation of transcription, DNA-templated	GO:0003677 DNA binding; GO:0003700 transcription factor activity, sequence-specific DNA; GO:0043565 sequence-specific DNA binding	GO:0005634 nucleus
Q00577.2	IPR006628	None predicted	None predicted	None predicted
Q01081.3	IPR009145; IPR000571; IPR012677; IPR000504; IPR003954	GO:0000398 mRNA splicing, via spliceosome	GO:0000166 nucleotide binding; GO:0003676 nucleic acid binding; GO:0003723 RNA binding; GO:0046872 metal ion binding	GO:0089701 U2AF
Q02108.2	IPR024096; IPR011644; IPR011645; IPR029787; IPR001054; IPR018297	GO:0006182 cGMP biosynthetic process; GO:0009190 cyclic nucleotide biosynthetic process; GO:0035556 intracellular signal transduction	GO:0004383 guanylate cyclase activity; GO:0016849 phosphorus-oxygen lyase activity; GO:0020037 heme binding	None predicted
Q03060.5	IPR001630; IPR003102; IPR004827	GO:0006355 regulation of transcription, DNA-templated	GO:0003677 DNA binding; GO:0003700 transcription factor activity, sequence-specific DNA; GO:0005515 protein binding; GO:0043565 sequence-specific DNA binding	GO:0005634 nucleus
Q03252.3	IPR001664; IPR001322; IPR018039	None predicted	GO:0005198 structural molecule activity	GO:0005882 intermediate filament
Q09MP3.1	IPR031419	None predicted	None predicted	None predicted
Q13033.3	IPR013258; IPR015943;	None predicted	GO:0005515 protein binding	None predicted

	IPR017986; IPR001680; IPR020472; IPR019775			
Q13111.2	IPR022043; IPR003917; IPR029091; IPR029105; IPR001750; IPR010933	GO:0006120 mitochondrial electron transport, NADH to ubiquinone; GO:0055114 oxidation-reduction process	GO:0008137 NADH dehydrogenase (ubiquinone) activity	None predicted
Q13114.2	IPR012227; IPR013083; IPR001841; IPR008974; IPR013323; IPR001293; IPR002083; IPR017907	GO:0007165 signal transduction; GO:0016567 protein ubiquitination; GO:0042981 regulation of apoptotic process	GO:0004842 ubiquitin-protein transferase activity; GO:0005515 protein binding; GO:0008270 zinc ion binding	None predicted
Q13118.1	IPR015880; IPR013087; IPR007087	None predicted	GO:0003676 nucleic acid binding; GO:0046872 metal ion binding	None predicted
Q13243.1	IPR012677; IPR000504	None predicted	GO:0000166 nucleotide binding; GO:0003676 nucleic acid binding	None predicted
Q13394.1	IPR024810	None predicted	None predicted	None predicted
Q15391.1	IPR000276; IPR005466; IPR017452	GO:0007186 G-protein coupled receptor signaling pathway	GO:0004930 G-protein coupled receptor activity; GO:0045028 G-protein coupled purinergic nucleotide receptor	GO:0016021 integral component of membrane
Q15573.1	IPR016629	GO:0006360 transcription from RNA polymerase I promoter	None predicted	GO:0000120 RNA polymerase I transcription factor complex
Q15629.3	IPR016447; IPR013599; IPR006634	None predicted	None predicted	GO:0016021 integral component of membrane
Q15695.2	IPR009145; IPR000571; IPR012677;	GO:0000398 mRNA splicing, via spliceosome	GO:0000166 nucleotide binding; GO:0003676 nucleic acid binding; GO:0003723 RNA binding;	GO:0089701 U2AF

	IPR000504; IPR003954		GO:0046872 metal ion binding	
Q16514.1	IPR009072; IPR003228	GO:0006352 DNA-templated transcription, initiation	GO:0046982 protein heterodimerization activity	GO:0005669 transcription factor TFIID complex
Q16560.1	IPR012677; IPR000504	None predicted	GO:0000166 nucleotide binding; GO:0003676 nucleic acid binding	None predicted
Q16763.2	IPR016135; IPR000608; IPR023313	None predicted	None predicted	None predicted
Q2I0M5.2	IPR009030; IPR006212; IPR000884	None predicted	None predicted	None predicted
Q2M296.2	IPR002698; IPR024185; IPR012677; IPR000504	None predicted	GO:0000166 nucleotide binding; GO:0003676 nucleic acid binding	None predicted
Q2TB18.1	IPR006085; IPR029060	GO:0006281 DNA repair	GO:0004518 nuclease activity	None predicted
Q3YEC7.2	IPR001806; IPR027417	GO:0007264 small GTPase mediated signal transduction	GO:0005525 GTP binding	None predicted
Q56UQ5.2	IPR018105; IPR011057; IPR011323; IPR018103	None predicted	None predicted	None predicted
Q5EBN2.1	IPR013083; IPR001841; IPR000315; IPR017907	None predicted	GO:0005515 protein binding; GO:0008270 zinc ion binding	GO:0005622 intracellular
Q5I0G3.1	IPR016040; IPR015955	GO:0005975 carbohydrate metabolic process; GO:0055114 oxidation-reduction process	GO:0003824 catalytic activity; GO:0016616 oxidoreductase activity, acting on the CH-OH group of	None predicted
Q5JPH6.2	IPR000924; IPR004527; IPR014729; IPR020058; IPR020061; IPR008925;	GO:0006418 tRNA aminoacylation for protein translation; GO:0006424 glutamyl-tRNA	GO:0000049 tRNA binding; GO:0000166 nucleotide binding; GO:0004812 aminoacyl-tRNA ligase activity; GO:0004818 glutamate-tRNA ligase activity; GO:0005524 ATP binding;	GO:0005737 cytoplasm

	IPR020751; IPR001412	aminoacylation; GO:0043039 tRNA aminoacylation	GO:0016876 ligase activity, forming aminoacyl-tRNA and related	
Q5JUK9.1	IPR031320	None predicted	None predicted	None predicted
Q5T3I0.2	IPR000467	None predicted	GO:0003676 nucleic acid binding	None predicted
Q5T8R8.1		None predicted	None predicted	None predicted
Q5TG30.3	IPR008936; IPR000198	GO:0007165 signal transduction	None predicted	None predicted
Q5VIR6.1	IPR007234	None predicted	None predicted	None predicted
Q6H8Q1.2	IPR001781; IPR032402; IPR003128	GO:0007010 cytoskeleton organization	GO:0003779 actin binding; GO:0008270 zinc ion binding	None predicted
Q6NT55.1	IPR001128; IPR002401; IPR017972	GO:0055114 oxidation-reduction process	GO:0005506 iron ion binding; GO:0016705 oxidoreductase activity, acting on paired donors, with; GO:0020037 heme binding	None predicted
Q6NWY9.1	IPR001202; IPR002713	None predicted	GO:0005515 protein binding	None predicted
Q6NXG1.2	IPR012337; IPR012677; IPR000504	None predicted	GO:0000166 nucleotide binding; GO:0003676 nucleic acid binding	None predicted
Q6PCT2.3	IPR002857; IPR013083; IPR011011; IPR001965; IPR019787; IPR001810; IPR032675; IPR006553; IPR019786	None predicted	GO:0003677 DNA binding; GO:0005515 protein binding; GO:0008270 zinc ion binding	None predicted
Q6Y288.2	IPR003378; IPR029044	None predicted	GO:0016757 transferase activity, transferring glycosyl groups	GO:0016020 membrane
Q7LGA3.1	IPR005331; IPR027417	None predicted	GO:0008146 sulfotransferase activity	GO:0016021 integral component of membrane

Q7Z422.1	IPR024771; IPR024642	None predicted	None predicted	None predicted
Q7Z5M5.3	IPR012496	None predicted	None predicted	GO:0016021 integral component of membrane
Q7Z5R6.1	IPR029071; IPR000159; IPR011993; IPR001849	GO:0007165 signal transduction	None predicted	None predicted
Q7Z6I6.3	IPR008936; IPR000198	GO:0007165 signal transduction	None predicted	None predicted
Q7Z7J5.2	IPR003034; IPR025892; IPR025891	None predicted	None predicted	None predicted
Q86UC2.1	IPR009290	None predicted	None predicted	None predicted
Q86UG4.2	IPR004156; IPR020846; IPR002350	GO:0006810 transport	GO:0005215 transporter activity; GO:0005515 protein binding	GO:0016020 membrane
Q86UT8.1	IPR028015	None predicted	None predicted	None predicted
Q86UX6.1	IPR011009; IPR000719; IPR017441; IPR008271	GO:0006468 protein phosphorylation	GO:0004672 protein kinase activity; GO:0005524 ATP binding	None predicted
Q8IV61.1	IPR000651; IPR023578; IPR001895; IPR011992; IPR002048; IPR020454; IPR002219; IPR018247	GO:0007264 small GTPase mediated signal transduction; GO:0035556 intracellular signal transduction	GO:0005085 guanyl-nucleotide exchange factor activity; GO:0005509 calcium ion binding	None predicted
Q8IV76.1	IPR000014	None predicted	None predicted	None predicted
Q8IX30.1	IPR000742; IPR001881; IPR009030; IPR024731; IPR026823; IPR011641; IPR000859;	None predicted	GO:0005509 calcium ion binding; GO:0005515 protein binding	None predicted

	IPR018097; IPR000152; IPR013032			
Q8IXL9.1	IPR000048	None predicted	GO:0005515 protein binding	None predicted
Q8IXR9.3	IPR027878	None predicted	None predicted	None predicted
Q8N1L4.2	IPR001128	GO:0055114 oxidation-reduction process	GO:0005506 iron ion binding; GO:0016705 oxidoreductase activity, acting on paired donors, with; GO:0020037 heme binding	None predicted
Q8N4E4.2	IPR012336; IPR024253	None predicted	None predicted	None predicted
Q8N4N8.3	IPR027417; IPR001752; IPR019821	GO:0007018 microtubule-based movement	GO:0003777 microtubule motor activity; GO:0005524 ATP binding; GO:0008017 microtubule binding	None predicted
Q8N609.2	IPR016447; IPR013599; IPR006634	None predicted	None predicted	GO:0016021 integral component of membrane
Q8N7E2.1	IPR013083; IPR001841; IPR007087; IPR017907	None predicted	GO:0005515 protein binding; GO:0008270 zinc ion binding; GO:0046872 metal ion binding	None predicted
Q8NB16.1	IPR011009; IPR000719; IPR001245	GO:0006468 protein phosphorylation	GO:0004672 protein kinase activity; GO:0005524 ATP binding	None predicted
Q8NBZ0.1		None predicted	None predicted	None predicted
Q8NEA5.1		None predicted	None predicted	None predicted
Q8NGW1.1	IPR000276; IPR000725; IPR017452	GO:0007186 G-protein coupled receptor signaling pathway	GO:0004930 G-protein coupled receptor activity; GO:0004984 olfactory receptor activity	GO:0016021 integral component of membrane
Q8NHX4.2	IPR026717	None predicted	None predicted	None predicted
Q8TDX6.2	IPR008428; IPR029044	None predicted	GO:0008376 acetylgalactosaminyltransferase activity	GO:0032580 Golgi cisterna membrane
Q8WU08.2	IPR011009; IPR000719;	GO:0006468 protein	GO:0004672 protein kinase	None

	IPR017441; IPR008271	phosphorylation	activity; GO:0005524 ATP binding	predicted
Q8WW32.2	IPR009071	None predicted	None predicted	None predicted
Q8WXA9.1	IPR012677; IPR000504	None predicted	GO:0000166 nucleotide binding; GO:0003676 nucleic acid binding	None predicted
Q8WXI4.1	IPR029069; IPR006683; IPR033120; IPR002913; IPR023393	None predicted	GO:0008289 lipid binding	None predicted
Q92187.1	IPR001675; IPR012163	GO:0006486 protein glycosylation	GO:0008373 sialyltransferase activity	GO:0030173 integral component of Golgi membrane
Q92599.4	IPR016491; IPR027417; IPR030379	None predicted	GO:0005525 GTP binding	None predicted
Q92665.3	IPR026299	None predicted	GO:0003735 structural constituent of ribosome	GO:0005763 mitochondrial small ribosomal subunit
Q92819.1	IPR029044; IPR001173	None predicted	None predicted	None predicted
Q92990.2	IPR013877	None predicted	None predicted	None predicted
Q96AQ8.1	IPR024461	None predicted	None predicted	None predicted
Q96BZ8.1	IPR019339	None predicted	None predicted	None predicted
Q96CM3.1	IPR020103; IPR006145; IPR006224	GO:0001522 pseudouridine synthesis; GO:0009451 RNA modification	GO:0003723 RNA binding; GO:0009982 pseudouridine synthase activity	None predicted
Q96HJ3.2		None predicted	None predicted	None predicted
Q96JC4.1	IPR001909; IPR015880; IPR013087;	GO:0006355 regulation of transcription,	GO:0003676 nucleic acid binding; GO:0046872 metal ion binding	GO:0005622 intracellular

	IPR007087	DNA-templated		
Q96LR9.2	IPR008405	GO:0006869 lipid transport; GO:0042157 lipoprotein metabolic process	GO:0008289 lipid binding	GO:0005576 extracellular region
Q96NF6.1		None predicted	None predicted	None predicted
Q96PB7.1	IPR011044; IPR022082; IPR003112	None predicted	None predicted	None predicted
Q99442.1	IPR004728; IPR011991	GO:0015031 protein transport	GO:0008565 protein transporter activity	GO:0016021 integral component of membrane
Q99558.2	IPR017425; IPR011009; IPR000719; IPR017441; IPR008271	GO:0006468 protein phosphorylation	GO:0004672 protein kinase activity; GO:0005524 ATP binding	None predicted
Q99680.2	IPR000276; IPR017452	GO:0007186 G-protein coupled receptor signaling pathway	GO:0004930 G-protein coupled receptor activity	GO:0016021 integral component of membrane
Q99683.1	IPR025136; IPR011009; IPR000719; IPR013761; IPR017441; IPR008271	GO:0006468 protein phosphorylation	GO:0004672 protein kinase activity; GO:0005524 ATP binding	None predicted
Q99784.4	IPR022082; IPR011044; IPR003112	None predicted	None predicted	None predicted
Q99999.1	IPR009729; IPR027417	GO:0009247 glycolipid biosynthetic process	GO:0001733 galactosylceramide sulfotransferase activity	GO:0005794 Golgi apparatus; GO:0016021 integral component of membrane
Q9BQG1.1	IPR000008; IPR001565	None predicted	GO:0005515 protein binding	GO:0016020 membrane

Q9BR76.1	IPR015048; IPR015943; IPR017986; IPR001680; IPR019775	None predicted	GO:0005515 protein binding	None predicted
Q9BRP8.1	IPR015362	None predicted	None predicted	None predicted
Q9BTM1.1	IPR002119; IPR009072; IPR007125; IPR032454; IPR032458	None predicted	GO:0003677 DNA binding; GO:0046982 protein heterodimerization activity	GO:0000786 nucleosome; GO:0005634 nucleus
Q9BUL5.1	IPR013083; IPR011011; IPR001965; IPR019787	None predicted	GO:0005515 protein binding; GO:0008270 zinc ion binding	None predicted
Q9BVV7.1	IPR013261	GO:0030150 protein import into mitochondrial matrix	None predicted	GO:0005744 mitochondrial inner membrane presequence translocase
Q9BY07.2	IPR003020; IPR003024; IPR013769; IPR016152; IPR011531	GO:0006810 transport; GO:0006820 anion transport	GO:0005215 transporter activity; GO:0005452 inorganic anion exchanger activity; GO:0008509 anion transmembrane transporter activity	GO:0016020 membrane; GO:0016021 integral component of membrane
Q9BY49.2	IPR002347; IPR016040	None predicted	None predicted	None predicted
Q9BZQ2.2	IPR006633; IPR011050; IPR012334; IPR006626	None predicted	None predicted	None predicted
Q9H000.2	IPR000571; IPR013083; IPR001841; IPR018957; IPR017907	None predicted	GO:0005515 protein binding; GO:0008270 zinc ion binding; GO:0046872 metal ion binding	None predicted
Q9H2G4.1	IPR002164	GO:0006334 nucleosome assembly	None predicted	GO:0005634 nucleus
Q9H974.1	IPR002616;	GO:0006400 tRNA modification;	GO:0008479 queuine tRNA-	None

	IPR028592	GO:0008616 queuosine biosynthetic process	ribosyltransferase activity	predicted
Q9HD33.2	IPR010729	GO:0006412 translation	GO:0003735 structural constituent of ribosome	GO:0005761 mitochondrial ribosome
Q9HD90.2	IPR016637; IPR011598; IPR022575	GO:0006355 regulation of transcription, DNA-templated; GO:0007399 nervous system development	GO:0046983 protein dimerization activity	None predicted
Q9NR83.4	IPR015880; IPR007087	None predicted	GO:0046872 metal ion binding	None predicted
Q9NSI5.2	IPR000996; IPR013783; IPR007110; IPR013098; IPR003599	GO:0006886 intracellular protein transport; GO:0016192 vesicle-mediated transport	GO:0005198 structural molecule activity; GO:0005515 protein binding	GO:0030130 clathrin coat of trans-Golgi network vesicle; GO:0030132 clathrin coat of coated pit
Q9NV88.2	IPR001279; IPR022712	None predicted	None predicted	None predicted
Q9NXH9.1	IPR002905; IPR029063; IPR000571	GO:0008033 tRNA processing	GO:0003723 RNA binding; GO:0004809 tRNA (guanine-N2-)-methyltransferase activity; GO:0046872 metal ion binding	None predicted
Q9NYB5.1	IPR004156; IPR020846; IPR002350	GO:0006810 transport	GO:0005215 transporter activity; GO:0005515 protein binding	GO:0016020 membrane
Q9NYL4.1	IPR001179	GO:0006457 protein folding	None predicted	None predicted
Q9P126.2	IPR016187; IPR016186; IPR001304	None predicted	None predicted	None predicted
Q9UKY7.1	IPR026806	None predicted	None predicted	None predicted
Q9ULL5.2	IPR025451	None predicted	None predicted	None predicted

Q9UPT9.2	IPR013083; IPR001607; IPR028889; IPR001394; IPR018200	GO:0006511 ubiquitin-dependent protein catabolic process; GO:0016579 protein deubiquitination	GO:0008270 zinc ion binding; GO:0036459 thiol-dependent ubiquitinyl hydrolase activity	None predicted
Q9UQ90.2	IPR005936; IPR011546; IPR027417; IPR003593; IPR003959; IPR000642	GO:0006508 proteolysis	GO:0004222 metalloendopeptidase activity; GO:0005524 ATP binding; GO:0008270 zinc ion binding	GO:0016020 membrane; GO:0016021 integral component of membrane
Q9Y324.1	IPR006984; IPR029060; IPR002716	None predicted	None predicted	GO:0032040 small-subunit processome
Q9Y383.2	IPR004882	GO:0006376 mRNA splice site selection	GO:0003729 mRNA binding	GO:0005685 U1 snRNP
Q9Y3C8.3	IPR014806; IPR016135	None predicted	None predicted	None predicted
Q9Y639.2	IPR016243; IPR013783; IPR007110; IPR003599; IPR003598	GO:0007169 transmembrane receptor protein tyrosine kinase signaling	GO:0004714 transmembrane receptor protein tyrosine kinase activity; GO:0005515 protein binding	None predicted

SUPPL TABLE S4. BCP NPC proteins and their predicted InterPro classification and GO categories

Protein	InterPro classification	GO (Biological Process)	GO (Molecular Function)	GO (Cellular Component)
A0FGR8.1	IPR031468; IPR000008	None predicted	GO:0005515 protein binding; GO:0008289 lipid binding	None predicted
A0JLT2.2	IPR019403	GO:0006357 regulation of transcription from RNA polymerase II	GO:0001104 RNA polymerase II transcription cofactor activity	GO:0016592 mediator complex
A2IDD5.1	IPR029329	None predicted	None predicted	None predicted
A6NCQ9.1	IPR013083; IPR001841; IPR027370; IPR017907	None predicted	GO:0005515 protein binding; GO:0008270 zinc ion binding	None predicted

A6NJT0.1	IPR009057; IPR001356; IPR017970	GO:0006355 regulation of transcription, DNA-templated	GO:0003677 DNA binding; GO:0043565 sequence-specific DNA binding	None predicted
A8MU76.2		None predicted	None predicted	None predicted
A8MV72.2		None predicted	None predicted	None predicted
A8MX80.2		None predicted	None predicted	None predicted
A8MZ26.2	IPR011992; IPR002048	None predicted	GO:0005509 calcium ion binding	None predicted
B4DS77.3	IPR026910	None predicted	None predicted	None predicted
E9PAV3.1		None predicted	None predicted	None predicted
F5H284.1	IPR024936; IPR029000; IPR002130; IPR020892	GO:0000413 protein peptidyl-prolyl isomerization; GO:0006457 protein folding	GO:0003755 peptidyl-prolyl cis-trans isomerase activity	None predicted
O00159.4	IPR027417; IPR001609; IPR010926; IPR000048	None predicted	GO:0003774 motor activity; GO:0005515 protein binding; GO:0005524 ATP binding	GO:0016459 myosin complex
O00401.2	IPR011993; IPR000697; IPR000095; IPR011026; IPR003124	GO:0007015 actin filament organization	GO:0003779 actin binding	None predicted
O00522.2	IPR032022; IPR020683; IPR019749; IPR000299; IPR014352; IPR019748; IPR002110	None predicted	GO:0005515 protein binding	GO:0005856 cytoskeleton
O14950.2	IPR011992; IPR002048; IPR015070; IPR018247	None predicted	GO:0005509 calcium ion binding	None predicted
O15234.2	IPR018545	None predicted	None predicted	None predicted
O15392.3	IPR001370	None predicted	None predicted	None predicted
O43189.3	IPR002999; IPR013083; IPR011011; IPR001965; IPR019787;	None predicted	GO:0005515 protein binding; GO:0008270 zinc ion binding	None predicted

	IPR025894; IPR019786			
O60504.2	IPR003127; IPR001452	None predicted	GO:0005515 protein binding	None predicted
O75084.2	IPR000539; IPR020067; IPR017981	GO:0007166 cell surface receptor signaling pathway	GO:0004888 transmembrane signaling receptor activity; GO:0005515 protein binding	GO:0016020 membrane
O75317.2	IPR028889; IPR001394; IPR018200	GO:0006511 ubiquitin-dependent protein catabolic process; GO:0016579 protein deubiquitination	GO:0036459 thiol-dependent ubiquitinyl hydrolase activity	None predicted
O75570.2	IPR005139; IPR014720; IPR000352	GO:0006415 translational termination	GO:0003747 translation release factor activity; GO:0016149 translation release factor activity, codon specific	GO:0005737 cytoplasm
O75912.1	IPR002219; IPR001206; IPR016064; IPR000756; IPR020683; IPR002110	GO:0007205 protein kinase C-activating G-protein coupled receptor; GO:0035556 intracellular signal transduction	GO:0004143 diacylglycerol kinase activity; GO:0005515 protein binding; GO:0016301 kinase activity	None predicted
O95236.3	IPR008405	GO:0006869 lipid transport; GO:0042157 lipoprotein metabolic process	GO:0008289 lipid binding	GO:0005576 extracellular region
O95260.2	IPR017137; IPR007471; IPR007472; IPR016181	GO:0016598 protein arginylation	GO:0004057 arginyltransferase activity	None predicted
O95456.1	IPR016565	GO:0043248 proteasome assembly	None predicted	GO:0005783 endoplasmic reticulum
O95837.1	IPR001019; IPR000654; IPR027417; IPR011025	GO:0007165 signal transduction; GO:0007186 G-protein coupled receptor signaling pathway	GO:0001664 G-protein coupled receptor binding; GO:0003924 GTPase activity; GO:0004871 signal transducer activity; GO:0005525 GTP binding; GO:0019001 guanyl nucleotide binding;	None predicted

			GO:0031683 G-protein beta/gamma-subunit complex binding	
O95886.3	IPR005026	GO:0023052 signaling	None predicted	None predicted
O96004.1	IPR011598	None predicted	GO:0046983 protein dimerization activity	None predicted
P05412.2	IPR002112; IPR005643; IPR008917; IPR004827	GO:0006355 regulation of transcription, DNA-templated	GO:0003677 DNA binding; GO:0003700 transcription factor activity, sequence-specific DNA; GO:0043565 sequence-specific DNA binding	None predicted
P05976.3	IPR011992; IPR002048	None predicted	GO:0005509 calcium ion binding	None predicted
P06858.1	IPR000734; IPR016272; IPR002330; IPR029058; IPR013818; IPR001024	GO:0006629 lipid metabolic process	GO:0004465 lipoprotein lipase activity; GO:0005515 protein binding; GO:0052689 carboxylic ester hydrolase activity	None predicted
P08588.2	IPR000276; IPR002233; IPR000507; IPR017452	GO:0007186 G-protein coupled receptor signaling pathway; GO:0007189 adenylate cyclase-activating G-protein coupled receptor; GO:0045823 positive regulation of heart contraction	GO:0004930 G-protein coupled receptor activity; GO:0004935 adrenergic receptor activity; GO:0004940 beta1-adrenergic receptor activity	GO:0016021 integral component of membrane
P11233.1	IPR001806; IPR020849; IPR027417; IPR005225	GO:0007165 signal transduction; GO:0007264 small GTPase mediated signal transduction	GO:0005525 GTP binding	GO:0016020 membrane
P14316.2	IPR017431; IPR011991; IPR001346; IPR019817	GO:0006355 regulation of transcription, DNA-templated	GO:0000975 regulatory region DNA binding; GO:0003700 transcription factor activity, sequence-specific DNA	None predicted
P14735.4	IPR011249; IPR011237; IPR011765;	GO:0006508 proteolysis	GO:0003824 catalytic activity; GO:0004222 metalloendopeptidase	None predicted

	IPR007863; IPR032632; IPR001431		activity; GO:0046872 metal ion binding	
P15692.2	IPR029034; IPR000072; IPR027928; IPR023581	None predicted	GO:0008083 growth factor activity; GO:0008201 heparin binding	GO:0016020 membrane
P17482.2	IPR017112; IPR006711; IPR009057; IPR001356; IPR020479; IPR017970	GO:0006351 transcription, DNA-templated; GO:0006355 regulation of transcription, DNA-templated	GO:0003677 DNA binding; GO:0043565 sequence-specific DNA binding	GO:0005634 nucleus
P17535.3	IPR002112; IPR005643; IPR008917; IPR004827	GO:0006355 regulation of transcription, DNA-templated	GO:0003677 DNA binding; GO:0003700 transcription factor activity, sequence-specific DNA; GO:0043565 sequence-specific DNA binding	None predicted
P19105.2	IPR011992; IPR002048; IPR015070; IPR018247	None predicted	GO:0005509 calcium ion binding	None predicted
P21728.1	IPR000276; IPR000929; IPR001413; IPR017452	GO:0007186 G-protein coupled receptor signaling pathway; GO:0010579 positive regulation of adenylate cyclase activity; GO:0042311 vasodilation	GO:0004930 G-protein coupled receptor activity; GO:0004952 dopamine neurotransmitter receptor activity	GO:0005887 integral component of plasma membrane; GO:0016021 integral component of membrane
P23526.4	IPR000043; IPR016040; IPR015878; IPR020082	GO:0006730 one-carbon metabolic process	GO:0004013 adenosylhomocysteinase activity	None predicted
P23942.1	IPR018499; IPR000830; IPR008952; IPR018498	GO:0007601 visual perception	None predicted	GO:0016021 integral component of membrane
P24844.4	IPR011992; IPR002048; IPR018247	None predicted	GO:0005509 calcium ion binding	None predicted

P29371.1	IPR000276; IPR001681; IPR001013; IPR017452	GO:0007186 G-protein coupled receptor signaling pathway	GO:0004930 G-protein coupled receptor activity; GO:0004995 tachykinin receptor activity	GO:0005886 plasma membrane; GO:0016021 integral component of membrane
P29466.1	IPR017350; IPR011029; IPR001315; IPR029030; IPR015917; IPR001309; IPR002138; IPR016129; IPR033139	GO:0006508 proteolysis; GO:0006915 apoptotic process; GO:0042981 regulation of apoptotic process	GO:0004197 cysteine-type endopeptidase activity; GO:0008234 cysteine-type peptidase activity	None predicted
P32754.2	IPR005956; IPR029068; IPR004360	GO:0009072 aromatic amino acid family metabolic process; GO:0055114 oxidation-reduction process	GO:0003868 4-hydroxyphenylpyruvate dioxygenase activity; GO:0016701 oxidoreductase activity, acting on single donors with	None predicted
P43487.1	IPR011993; IPR000156	GO:0046907 intracellular transport	None predicted	None predicted
P46777.3	IPR005485; IPR025607	GO:0006412 translation	GO:0003735 structural constituent of ribosome; GO:0008097 5S rRNA binding	GO:0005622 intracellular; GO:0005840 ribosome
P48163.1	IPR001891; IPR012301; IPR016040; IPR012302; IPR015884	GO:0006108 malate metabolic process; GO:0055114 oxidation-reduction process	GO:0004470 malic enzyme activity; GO:0004471 malate dehydrogenase (decarboxylating) (NAD+) activity; GO:0051287 NAD binding	None predicted
P48167.1	IPR006201; IPR008060; IPR006202; IPR006029; IPR018000	GO:0006810 transport; GO:0006811 ion transport; GO:0006821 chloride transport	GO:0005230 extracellular ligand-gated ion channel activity; GO:0016934 extracellular-glycine-gated chloride channel activity	GO:0016020 membrane; GO:0016021 integral component of membrane; GO:0045211 postsynaptic membrane

P48745.1	IPR012395; IPR009030; IPR000867; IPR001007; IPR006208; IPR006207; IPR000884; IPR017891	GO:0001558 regulation of cell growth	GO:0005515 protein binding; GO:0005520 insulin-like growth factor binding	GO:0005576 extracellular region
P48751.2	IPR003020; IPR001717; IPR002979; IPR016152; IPR013769; IPR011531; IPR018241	GO:0006810 transport; GO:0006820 anion transport	GO:0005215 transporter activity; GO:0005452 inorganic anion exchanger activity; GO:0008509 anion transmembrane transporter activity	GO:0016020 membrane; GO:0016021 integral component of membrane
P48995.1	IPR004729; IPR002153; IPR005457; IPR020683; IPR013555; IPR005821; IPR002110	GO:0006811 ion transport; GO:0055085 transmembrane transport; GO:0070588 calcium ion transmembrane transport	GO:0005216 ion channel activity; GO:0005262 calcium channel activity; GO:0005515 protein binding	GO:0016020 membrane; GO:0016021 integral component of membrane
P49757.2	IPR016698; IPR011993; IPR006020; IPR010449	None predicted	GO:0005515 protein binding	None predicted
P51815.2	IPR008916; IPR003309; IPR001909; IPR015880; IPR013087; IPR007087	GO:0006355 regulation of transcription, DNA-templated	GO:0003676 nucleic acid binding; GO:0003700 transcription factor activity, sequence-specific DNA; GO:0046872 metal ion binding	GO:0005622 intracellular
P60059.1	IPR001901; IPR008158; IPR022943; IPR023391	GO:0006605 protein targeting; GO:0006886 intracellular protein transport; GO:0015031 protein transport	GO:0015450 P-P-bond-hydrolysis-driven protein transmembrane	GO:0016020 membrane
P60608.1	IPR018154	None predicted	None predicted	None predicted
P61201.1	IPR011990; IPR000717; IPR011991	None predicted	GO:0005515 protein binding	None predicted
P61758.3	IPR009053; IPR004127; IPR016655	GO:0006457 protein folding	None predicted	GO:0016272 prefoldin complex

P62314.1	IPR010920; IPR001163	None predicted	None predicted	None predicted
Q00444.1	IPR017995; IPR009057; IPR001356; IPR020479; IPR001827; IPR017970	GO:0006355 regulation of transcription, DNA-templated	GO:0003677 DNA binding; GO:0003700 transcription factor activity, sequence-specific DNA; GO:0043565 sequence-specific DNA binding	GO:0005634 nucleus
Q01081.3	IPR009145; IPR000571; IPR012677; IPR000504; IPR003954	GO:0000398 mRNA splicing, via spliceosome	GO:0000166 nucleotide binding; GO:0003676 nucleic acid binding; GO:0003723 RNA binding; GO:0046872 metal ion binding	GO:0089701 U2AF
Q02108.2	IPR024096; IPR011644; IPR011645; IPR029787; IPR001054; IPR018297	GO:0006182 cGMP biosynthetic process; GO:0009190 cyclic nucleotide biosynthetic process; GO:0035556 intracellular signal transduction	GO:0004383 guanylate cyclase activity; GO:0016849 phosphorus-oxygen lyase activity; GO:0020037 heme binding	None predicted
Q03060.5	IPR001630; IPR003102; IPR004827	GO:0006355 regulation of transcription, DNA-templated	GO:0003677 DNA binding; GO:0003700 transcription factor activity, sequence-specific DNA; GO:0005515 protein binding; GO:0043565 sequence-specific DNA binding	GO:0005634 nucleus
Q03591.2	IPR000436	None predicted	None predicted	None predicted
Q05215.3	IPR015880; IPR013087; IPR007087	None predicted	GO:0003676 nucleic acid binding; GO:0046872 metal ion binding	None predicted
Q06416.2	IPR010982; IPR000327; IPR013847; IPR009057; IPR001356; IPR017970	GO:0006355 regulation of transcription, DNA-templated	GO:0003677 DNA binding; GO:0003700 transcription factor activity, sequence-specific DNA; GO:0043565 sequence-specific DNA binding	None predicted
Q08379.3	IPR024858	None predicted	None predicted	GO:0005794 Golgi apparatus
Q13033.3	IPR013258; IPR015943;	None predicted	GO:0005515 protein binding	None predicted

	IPR017986; IPR001680; IPR020472; IPR019775			
Q13454.1	IPR021149; IPR012336	None predicted	None predicted	None predicted
Q13562.3	IPR016637; IPR011598; IPR022575	GO:0006355 regulation of transcription, DNA-templated; GO:0007399 nervous system development	GO:0046983 protein dimerization activity	None predicted
Q14332.1	IPR000539; IPR020067; IPR017981	GO:0007166 cell surface receptor signaling pathway	GO:0004888 transmembrane signaling receptor activity; GO:0005515 protein binding	GO:0016020 membrane
Q15404.3	IPR032675; IPR003591; IPR001611	None predicted	GO:0005515 protein binding	None predicted
Q15629.3	IPR016447; IPR013599; IPR006634	None predicted	None predicted	GO:0016021 integral component of membrane
Q15645.2	IPR001270; IPR027417; IPR003593; IPR003959; IPR003960	None predicted	GO:0005524 ATP binding	None predicted
Q16206.2	IPR012677; IPR000504	None predicted	GO:0000166 nucleotide binding; GO:0003676 nucleic acid binding	None predicted
Q16763.2	IPR016135; IPR000608; IPR023313	None predicted	None predicted	None predicted
Q49AM3.3	IPR011990; IPR013026; IPR019734	None predicted	GO:0005515 protein binding	None predicted
Q52LC2.1	IPR024722	None predicted	None predicted	None predicted
Q5EG05.1	IPR011029; IPR001315	GO:0042981 regulation of apoptotic process	None predicted	None predicted
Q5HYW3.1	IPR032549	None predicted	None predicted	None predicted
Q5JTH9.2	IPR016024;	None predicted	GO:0005488 binding	None predicted

	IPR011989; IPR012978			
Q5JUK9.1	IPR000883; IPR031320; IPR023616; IPR023615	GO:0009060 aerobic respiration; GO:0055114 oxidation-reduction process	GO:0004129 cytochrome-c oxidase activity; GO:0005506 iron ion binding; GO:0009055 electron carrier activity; GO:0020037 heme binding	GO:0016021 integral component of membrane
Q5SY16.1	IPR032319	None predicted	None predicted	None predicted
Q5T447.1	IPR008979; IPR004939; IPR000569	None predicted	GO:0004842 ubiquitin-protein transferase activity	None predicted
Q5T4B2.1	IPR002654; IPR029044	None predicted	None predicted	None predicted
Q5VZ72.4	IPR029389; IPR001128	GO:0055114 oxidation-reduction process	GO:0005506 iron ion binding; GO:0016705 oxidoreductase activity, acting on paired donors, with; GO:0020037 heme binding	None predicted
Q68DH5.1	IPR006876	None predicted	None predicted	None predicted
Q69YI7.1	IPR028002	None predicted	None predicted	None predicted
Q6AZZ1.1	IPR013083; IPR001841; IPR000315; IPR001870; IPR013320; IPR003879; IPR006574; IPR003877; IPR017907	None predicted	GO:0005515 protein binding; GO:0008270 zinc ion binding	GO:0005622 intracellular
Q6PGP7.1	IPR013026; IPR011990; IPR019734	None predicted	GO:0005515 protein binding	None predicted
Q6PIU1.2	IPR028325; IPR003968; IPR003970; IPR011333; IPR000210; IPR003131; IPR027359; IPR005821	GO:0006811 ion transport; GO:0006813 potassium ion transport; GO:0051260 protein homooligomerization; GO:0055085 transmembrane transport	GO:0005216 ion channel activity; GO:0005249 voltage-gated potassium channel activity; GO:0005515 protein binding	GO:0008076 voltage-gated potassium channel complex; GO:0016020 membrane

Q6STE5.1	IPR003121; IPR019835	None predicted	GO:0005515 protein binding	None predicted
Q6XYQ8.1	IPR000008; IPR001565	None predicted	GO:0005515 protein binding	GO:0016020 membrane
Q6Y288.2	IPR003378; IPR029044	None predicted	GO:0016757 transferase activity, transferring glycosyl groups	GO:0016020 membrane
Q6ZW13.2	IPR031516	None predicted	None predicted	None predicted
Q75V66.1	IPR007632; IPR032394	None predicted	GO:0046983 protein dimerization activity	None predicted
Q7Z3Y9.2	IPR001664; IPR002957	None predicted	GO:0005198 structural molecule activity	GO:0005882 intermediate filament
Q7Z422.1	IPR024771; IPR024642	None predicted	None predicted	None predicted
Q7Z4G4.1	IPR016691; IPR029063; IPR000241; IPR002052	GO:0032259 methylation	GO:0003676 nucleic acid binding; GO:0008168 methyltransferase activity	None predicted
Q7Z5R6.1	IPR029071; IPR000159; IPR011993; IPR001849	GO:0007165 signal transduction	None predicted	None predicted
Q7Z7J5.2	IPR003034; IPR025892; IPR025891	None predicted	None predicted	None predicted
Q86W34.2	IPR012962; IPR024079	None predicted	GO:0008237 metallopeptidase activity; GO:0008270 zinc ion binding	None predicted
Q86XJ1.1	IPR001715; IPR003108	None predicted	GO:0005515 protein binding; GO:0008017 microtubule binding	None predicted
Q8IYV9.2	IPR029389; IPR013783; IPR007110; IPR032699	None predicted	GO:0005515 protein binding	None predicted
Q8N609.2	IPR016447; IPR013599; IPR006634	None predicted	None predicted	GO:0016021 integral component of membrane

Q8N7X8.1	IPR013783; IPR007110	None predicted	GO:0005515 protein binding	None predicted
Q8NB90.3	IPR009010; IPR027417; IPR003593; IPR003959; IPR003960	None predicted	GO:0005524 ATP binding	None predicted
Q8NBZ0.1		None predicted	None predicted	None predicted
Q8NEC5.3	IPR027359; IPR005821	GO:0006811 ion transport; GO:0055085 transmembrane transport	GO:0005216 ion channel activity	GO:0016020 membrane
Q8NHX4.2	IPR026717	None predicted	None predicted	None predicted
Q8TAF7.2	IPR001909; IPR015880; IPR007087; IPR013087	GO:0006355 regulation of transcription, DNA-templated	GO:0003676 nucleic acid binding; GO:0046872 metal ion binding	GO:0005622 intracellular
Q8TE04.2	IPR004567; IPR000440	GO:0015937 coenzyme A biosynthetic process; GO:0055114 oxidation-reduction process	GO:0004594 pantothenate kinase activity; GO:0005524 ATP binding; GO:0008137 NADH dehydrogenase (ubiquinone) activity	None predicted
Q8TF01.2	IPR031937	None predicted	None predicted	None predicted
Q8WU08.2	IPR011009; IPR000719; IPR017441; IPR008271	GO:0006468 protein phosphorylation	GO:0004672 protein kinase activity; GO:0005524 ATP binding	None predicted
Q8WUN7.2	IPR032752; IPR029071; IPR000626	None predicted	GO:0005515 protein binding; GO:0043130 ubiquitin binding	None predicted
Q92598.1	IPR013126; IPR029047; IPR029048; IPR018181	None predicted	None predicted	None predicted
Q92623.3	IPR011990; IPR013026; IPR019734; IPR013105	None predicted	GO:0005515 protein binding	None predicted
Q92670.2	IPR008916; IPR003309; IPR001909; IPR015880;	GO:0006355 regulation of transcription, DNA-templated	GO:0003676 nucleic acid binding; GO:0003700 transcription factor activity, sequence-specific	GO:0005622 intracellular

	IPR013087; IPR007087		DNA; GO:0046872 metal ion binding	
Q969X2.1	IPR001675	GO:0006486 protein glycosylation	GO:0008373 sialyltransferase activity	None predicted
Q96AN5.1	IPR022227	None predicted	None predicted	None predicted
Q96AQ8.1	IPR024461	None predicted	None predicted	None predicted
Q96C57.2		None predicted	None predicted	None predicted
Q96EK5.1	IPR022083; IPR011990	None predicted	GO:0005515 protein binding	None predicted
Q96IC2.1	IPR012337; IPR013520; IPR012677; IPR000504	None predicted	GO:0000166 nucleotide binding; GO:0003676 nucleic acid binding	None predicted
Q96KK3.2	IPR028325; IPR003968; IPR003971; IPR011333; IPR000210; IPR003131; IPR027359; IPR005821	GO:0006811 ion transport; GO:0006813 potassium ion transport; GO:0051260 protein homooligomerization; GO:0055085 transmembrane transport	GO:0005216 ion channel activity; GO:0005249 voltage-gated potassium channel activity; GO:0005515 protein binding	GO:0008076 voltage-gated potassium channel complex; GO:0016020 membrane
Q96N20.1	IPR001909; IPR015880; IPR013087; IPR007087	GO:0006355 regulation of transcription, DNA-templated	GO:0003676 nucleic acid binding; GO:0046872 metal ion binding	GO:0005622 intracellular
Q96NR3.2	IPR003392; IPR000731	None predicted	None predicted	GO:0016021 integral component of membrane
Q99558.2	IPR017425; IPR011009; IPR000719; IPR017441; IPR008271	GO:0006468 protein phosphorylation	GO:0004672 protein kinase activity; GO:0005524 ATP binding	None predicted
Q99578.1	IPR001806; IPR020849; IPR027417; IPR005225	GO:0007165 signal transduction; GO:0007264 small GTPase mediated signal transduction	GO:0005525 GTP binding	GO:0016020 membrane
Q99608.1	IPR002190	None predicted	None predicted	None predicted

Q99626.3	IPR006820; IPR009057; IPR001356; IPR020479; IPR000047; IPR017970	GO:0006355 regulation of transcription, DNA-templated; GO:0007275 multicellular organism development	GO:0003677 DNA binding; GO:0043565 sequence-specific DNA binding	GO:0005634 nucleus
Q99680.2	IPR000276; IPR017452	GO:0007186 G-protein coupled receptor signaling pathway	GO:0004930 G-protein coupled receptor activity	GO:0016021 integral component of membrane
Q9BQ15.1	IPR012340; IPR004365	None predicted	GO:0003676 nucleic acid binding	None predicted
Q9BT49.2	IPR006612	None predicted	GO:0003676 nucleic acid binding	None predicted
Q9BXF9.1	IPR000435	None predicted	None predicted	None predicted
Q9BY07.2	IPR003020; IPR003024; IPR013769; IPR016152; IPR011531	GO:0006810 transport; GO:0006820 anion transport	GO:0005215 transporter activity; GO:0005452 inorganic anion exchanger activity; GO:0008509 anion transmembrane transporter activity	GO:0016020 membrane; GO:0016021 integral component of membrane
Q9BYE3.1	IPR028205	GO:0008544 epidermis development	None predicted	None predicted
Q9H1X3.1	IPR001623	None predicted	None predicted	None predicted
Q9H239.2	IPR021190; IPR016293; IPR002477; IPR024079; IPR006026; IPR001818; IPR033739; IPR000585; IPR018487	GO:0006508 proteolysis	GO:0004222 metalloendopeptidase activity; GO:0005509 calcium ion binding; GO:0008237 metallopeptidase activity; GO:0008270 zinc ion binding	GO:0031012 extracellular matrix
Q9H2G4.1	IPR002164	GO:0006334 nucleosome assembly	None predicted	GO:0005634 nucleus
Q9H6E4.1	IPR026321	None predicted	None predicted	None predicted
Q9H6Y7.1	IPR003137; IPR013083; IPR001841; IPR011016	None predicted	GO:0005515 protein binding; GO:0008270 zinc ion binding	None predicted
Q9H813.1	IPR029366	None predicted	None predicted	None predicted

Q9HB58.5	IPR004865; IPR010919; IPR000770; IPR013083; IPR011011; IPR001965; IPR019787; IPR001487; IPR019786	None predicted	GO:0003677 DNA binding; GO:0005515 protein binding; GO:0008270 zinc ion binding	GO:0005634 nucleus
Q9HCP0.1	IPR011009; IPR000719; IPR022247; IPR017441; IPR008271	GO:0006468 protein phosphorylation	GO:0004672 protein kinase activity; GO:0004674 protein serine/threonine kinase activity; GO:0005524 ATP binding	None predicted
Q9NRQ5.1	IPR027960	None predicted	None predicted	None predicted
Q9NS84.2	IPR016469; IPR027417; IPR000863	GO:0005975 carbohydrate metabolic process	GO:0008146 sulfotransferase activity	GO:0000139 Golgi membrane
Q9NZE8.3	IPR021137	GO:0006412 translation	GO:0003735 structural constituent of ribosome	GO:0005622 intracellular; GO:0005840 ribosome
Q9NZM6.2	IPR003915; IPR013122	None predicted	GO:0005509 calcium ion binding	GO:0016020 membrane
Q9NZU5.1	IPR010442; IPR033724; IPR001781	None predicted	GO:0008270 zinc ion binding	None predicted
Q9P0P8.1		None predicted	None predicted	None predicted
Q9P215.2	IPR001909; IPR018586; IPR009057; IPR006600; IPR004875	GO:0006355 regulation of transcription, DNA-templated	GO:0003676 nucleic acid binding; GO:0003677 DNA binding	GO:0005622 intracellular
Q9P2K5.3	IPR012677; IPR000504	None predicted	GO:0000166 nucleotide binding; GO:0003676 nucleic acid binding	None predicted
Q9UJK0.1	IPR022968; IPR007209; IPR007177	None predicted	None predicted	None predicted
Q9UJT0.1	IPR000217; IPR004057; IPR003008; IPR018316;	GO:0007017 microtubule-based process	GO:0003924 GTPase activity; GO:0005525 GTP binding	GO:0005874 microtubule

	IPR008280; IPR023123; IPR017975			
Q9UKG4.2	IPR001898	GO:0006814 sodium ion transport; GO:0055085 transmembrane transport	GO:0005215 transporter activity	GO:0016020 membrane
Q9UM00.1	IPR002809; IPR008559	None predicted	None predicted	GO:0016020 membrane
Q9UPG8.1	IPR015880; IPR013087; IPR007087	None predicted	GO:0003676 nucleic acid binding; GO:0046872 metal ion binding	None predicted
Q9UPT9.2	IPR013083; IPR001607; IPR028889; IPR001394; IPR018200	GO:0006511 ubiquitin-dependent protein catabolic process; GO:0016579 protein deubiquitination	GO:0008270 zinc ion binding; GO:0036459 thiol-dependent ubiquitinyl hydrolase activity	None predicted
Q9UQ90.2	IPR005936; IPR011546; IPR027417; IPR003593; IPR003959; IPR000642	GO:0006508 proteolysis	GO:0004222 metalloendopeptidase activity; GO:0005524 ATP binding; GO:0008270 zinc ion binding	GO:0016020 membrane; GO:0016021 integral component of membrane
Q9Y2B1.1	IPR004263	None predicted	None predicted	None predicted
Q9Y3M8.2	IPR013761; IPR001660; IPR008936; IPR000198; IPR002913; IPR023393	GO:0007165 signal transduction	GO:0005515 protein binding; GO:0008289 lipid binding	None predicted
Q9Y3N9.1	IPR000276; IPR000725; IPR017452	GO:0007186 G-protein coupled receptor signaling pathway	GO:0004930 G-protein coupled receptor activity; GO:0004984 olfactory receptor activity	GO:0016021 integral component of membrane
Q9Y5Q3.2	IPR013592; IPR008917; IPR004826; IPR004827	GO:0006355 regulation of transcription, DNA-templated	GO:0003677 DNA binding; GO:0003700 transcription factor activity, sequence-specific DNA; GO:0043565 sequence-specific DNA binding	GO:0005634 nucleus

SUPPL TABLE S5 Summary list of Cellular Components GO terms based on the functional enrichment analysis results for NPC proteins in the HF and BCP blood

GO categories "Cellular components"	HFs	BCPs
GO:0000120 RNA polymerase I transcription factor complex	1	-
GO:0000139 Golgi membrane	-	1
GO:0000786 nucleosome	1	-
GO:0005576 extracellular region	3	2
GO:0005622 intracellular	2	8
GO:0005634 nucleus	10	7
GO:0005669 transcription factor TFIID complex	1	-
GO:0005685 U1 snRNP	1	-
GO:0005737 cytoplasm	4	1
GO:0005744 mitochondrial inner membrane presequence translocase	1	-
GO:0005761 mitochondrial ribosome	1	-
GO:0005763 mitochondrial small ribosomal subunit	1	-
GO:0005783 endoplasmic reticulum	-	1
GO:0005794 Golgi apparatus	1	1
GO:0005840 ribosome	-	2
GO:0005856 cytoskeleton	1	1
GO:0005874 microtubule	-	1
GO:0005882 intermediate filament	1	1
GO:0005886 plasma membrane	-	1
GO:0005887 integral component of plasma membrane	1	1
GO:0008076 voltage-gated potassium channel complex	-	2
GO:0016020 membrane	10	19
GO:0016021 integral component of membrane	15	15
GO:0016272 prefoldin complex	-	1
GO:0016459 myosin complex	-	1
GO:0016471 vacuolar proton-transporting V-type ATPase complex	1	-
GO:0016592 mediator complex	-	1
GO:0030130 clathrin coat of trans-Golgi network vesicle	1	-
GO:0030132 clathrin coat of coated pit	1	-
GO:0030173 integral component of Golgi membrane	1	-
GO:0031012 extracellular matrix	-	1
GO:0032040 small-subunit processome	1	-
GO:0032580 Golgi cisterna membrane	1	-
GO:0042555 MCM complex	1	-
GO:0045211 postsynaptic membrane	1	1

GO:0089701 U2AF	2	1
Total GO: 36 (100%)	26 (72%)	22 (61%)
Total proteins:	65	70

SUPPL TABLE S6 Summary list of Molecular Function GO terms based on the functional enrichment analysis results for NPC proteins in the HF and BCP blood

GO categories " Molecular Functions"	HFs	BCPs
GO:0000049 tRNA binding	1	-
GO:0000166 nucleotide binding	9	4
GO:0000975 regulatory region DNA binding	-	1
GO:0001104 RNA polymerase II transcription cofactor activity	-	1
GO:0001664 G-protein coupled receptor binding	-	1
GO:0001733 galactosylceramide sulfotransferase activity	1	-
GO:0003676 nucleic acid binding	10	14
GO:0003677 DNA binding	10	11
GO:0003678 DNA helicase activity	1	-
GO:0003700 transcription factor activity, sequence-specific DNA	5	9
GO:0003723 RNA binding	4	1
GO:0003729 mRNA binding	1	-
GO:0003735 structural constituent of ribosome	2	2
GO:0003743 translation initiation factor activity	1	-
GO:0003747 translation release factor activity	1	1
GO:0003755 peptidyl-prolyl cis-trans isomerase activity	-	1
GO:0003774 motor activity	-	1
GO:0003777 microtubule motor activity	1	-
GO:0003779 actin binding	2	1
GO:0003824 catalytic activity	4	1
GO:0003868 4-hydroxyphenylpyruvate dioxygenase activity	-	1
GO:0003883 CTP synthase activity	1	-
GO:0003924 GTPase activity	1	2
GO:0004013 adenosylhomocysteinase activity	-	1
GO:0004057 arginyltransferase activity	-	1

GO:0004129 cytochrome-c oxidase activity	-	1
GO:0004143 diacylglycerol kinase activity	-	1
GO:0004197 cysteine-type endopeptidase activity	-	1
GO:0004222 metalloendopeptidase activity	2	3
GO:0004252 serine-type endopeptidase activity	2	-
GO:0004347 glucose-6-phosphate isomerase activity	1	-
GO:0004372 glycine hydroxymethyltransferase activity	1	-
GO:0004383 guanylate cyclase activity	1	1
GO:0004402 histone acetyltransferase activity	1	-
GO:0004465 lipoprotein lipase activity	-	1
GO:0004470 malic enzyme activity	1	1
GO:0004471 malate dehydrogenase (decarboxylating) (NAD+) activity	1	1
GO:0004518 nuclease activity	1	-
GO:0004594 pantothenate kinase activity	-	1
GO:0004672 protein kinase activity	7	3
GO:0004674 protein serine/threonine kinase activity	1	1
GO:0004714 transmembrane receptor protein tyrosine kinase activity	1	-
GO:0004809 tRNA (guanine-N2-)-methyltransferase activity	1	-
GO:0004812 aminoacyl-tRNA ligase activity	2	-
GO:0004818 glutamate-tRNA ligase activity	1	-
GO:0004829 threonine-tRNA ligase activity	1	-
GO:0004842 ubiquitin-protein transferase activity	1	1
GO:0004871 signal transducer activity	-	1
GO:0004888 transmembrane signaling receptor activity	-	2
GO:0004930 G-protein coupled receptor activity	6	5
GO:0004935 adrenergic receptor activity	2	1
GO:0004938 alpha2-adrenergic receptor activity	1	-
GO:0004940 beta1-adrenergic receptor activity	1	1
GO:0004952 dopamine neurotransmitter receptor activity	-	1
GO:0004984 olfactory receptor activity	1	1
GO:0004990 oxytocin receptor activity	1	-

GO:0004995 tachykinin receptor activity	-	1
GO:0005000 vasopressin receptor activity	1	-
GO:0005085 guanyl-nucleotide exchange factor activity	1	-
GO:0005089 Rho guanyl-nucleotide exchange factor activity	1	-
GO:0005198 structural molecule activity	2	1
GO:0005215 transporter activity	3	3
GO:0005216 ion channel activity	1	4
GO:0005230 extracellular ligand-gated ion channel activity	1	1
GO:0005249 voltage-gated potassium channel activity	-	2
GO:0005262 calcium channel activity	-	1
GO:0005452 inorganic anion exchanger activity	1	2
GO:0005488 binding	1	1
GO:0005506 iron ion binding	2	2
GO:0005509 calcium ion binding	6	7
GO:0005515 protein binding	25	33
GO:0005520 insulin-like growth factor binding	1	1
GO:0005524 ATP binding	13	8
GO:0005525 GTP binding	6	4
GO:0008017 microtubule binding	1	1
GO:0008083 growth factor activity	-	1
GO:0008097 5S rRNA binding	-	1
GO:0008137 NADH dehydrogenase (ubiquinone) activity	-	1
GO:0008146 sulfotransferase activity	-	1
GO:0008168 methyltransferase activity	-	1
GO:0008137 NADH dehydrogenase (ubiquinone) activity	1	-
GO:0008146 sulfotransferase activity	1	-
GO:0008201 heparin binding	-	1
GO:0008234 cysteine-type peptidase activity	-	1
GO:0008237 metallopeptidase activity	-	2
GO:0008270 zinc ion binding	12	10
GO:0008289 lipid binding	2	3

GO:0008373 sialyltransferase activity	1	1
GO:0008376 acetylgalactosaminyltransferase activity	1	-
GO:0008479 queuine tRNA-ribosyltransferase activity	1	-
GO:0008509 anion transmembrane transporter activity	1	2
GO:0008565 protein transporter activity	1	-
GO:0009055 electron carrier activity	-	1
GO:0009982 pseudouridine synthase activity	1	-
GO:0015450 P-P-bond-hydrolysis-driven protein transmembrane	-	1
GO:0016149 translation release factor activity, codon specific	1	1
GO:0016301 kinase activity	-	1
GO:0016594 glycine binding	1	-
GO:0016616 oxidoreductase activity, acting on the CH-OH group	1	-
GO:0016701 oxidoreductase activity, acting on single donors	-	1
GO:0016705 oxidoreductase activity, acting on paired donors	2	1
GO:0016740 transferase activity	1	-
GO:0016757 transferase activity, transferring glycosyl groups	1	1
GO:0016758 transferase activity, transferring hexosyl groups	1	-
GO:0016787 hydrolase activity	1	-
GO:0016820 hydrolase activity, acting on acid anhydrides,	1	-
GO:0016849 phosphorus-oxygen lyase activity	1	1
GO:0016876 ligase activity, forming aminoacyl-tRNA and related	2	-
GO:0016934 extracellular-glycine-gated chloride channel activity	1	1
GO:0019001 guanyl nucleotide binding	-	1
GO:0020037 heme binding	3	3
GO:0022824 transmitter-gated ion channel activity	1	-
GO:0030170 pyridoxal phosphate binding	2	-
GO:0031683 G-protein beta/gamma-subunit complex binding	-	1
GO:0036459 thiol-dependent ubiquitinyl hydrolase activity	2	2
GO:0043130 ubiquitin binding	-	1
GO:0043565 sequence-specific DNA binding	6	9
GO:0045028 G-protein coupled purinergic nucleotide receptor	1	-

GO:0045182 translation regulator activity	1	-
GO:0046872 metal ion binding	10	8
GO:0046982 protein heterodimerization activity	2	-
GO:0046983 protein dimerization activity	2	3
GO:0051082 unfolded protein binding	1	-
GO:0051287 NAD binding	1	1
GO:0052689 carboxylic ester hydrolase activity	-	1
Total GO: 125	90 (72%)	84 (67%)
Total proteins:	227	218

SUPPL TABLE S7 Summary list of Biological Processes GO terms based on the functional enrichment analysis results for NPC proteins in the HF and BCP blood

GO categories Biological processes "	HFs	BCPs
GO:0000398 mRNA splicing, via spliceosome	2	1
GO:0000413 protein peptidyl-prolyl isomerization; GO:0006457 protein folding	-	1
GO:0000902 cell morphogenesis	1	-
GO:0001522 pseudouridine synthesis	1	-
GO:0001558 regulation of cell growth	1	1
GO:0005975 carbohydrate metabolic process	1	1
GO:0006094 gluconeogenesis	1	-
GO:0006096 glycolytic process	1	-
GO:0006108 malate metabolic process	1	1
GO:0006120 mitochondrial electron transport, NADH to ubiquinone	1	-
GO:0006182 cGMP biosynthetic process	1	1
GO:0006221 pyrimidine nucleotide biosynthetic process	1	-
GO:0006260 DNA replication	1	-
GO:0006270 DNA replication initiation	1	-
GO:0006281 DNA repair	1	-
GO:0006334 nucleosome assembly	1	1
GO:0006348 chromatin silencing at telomere	1	-
GO:0006351 transcription, DNA-templated	-	1

GO:0006352 DNA-templated transcription, initiation	1	-
GO:0006355 regulation of transcription, DNA-templated	8	16
GO:0006357 regulation of transcription from RNA polymerase II	-	1
GO:0006360 transcription from RNA polymerase I promoter	1	-
GO:0006376 mRNA splice site selection	1	-
GO:0006400 tRNA modification	1	-
GO:0006412 translation	1	2
GO:0006413 translational initiation	1	-
GO:0006415 translational termination	1	1
GO:0006418 tRNA aminoacylation for protein translation	2	-
GO:0006424 glutamyl-tRNA aminoacylation	1	-
GO:0006435 threonyl-tRNA aminoacylation	1	-
GO:0006457 protein folding	2	1
GO:0006468 protein phosphorylation	7	3
GO:0006486 protein glycosylation	1	1
GO:0006508 proteolysis	4	4
GO:0006511 ubiquitin-dependent protein catabolic process	2	2
GO:0006544 glycine metabolic process	1	-
GO:0006563 L-serine metabolic process	1	-
GO:0006605 protein targeting	-	1
GO:0006629 lipid metabolic process	-	1
GO:0006730 one-carbon metabolic process	-	1
GO:0006810 transport	4	3
GO:0006811 ion transport	2	5
GO:0006813 potassium ion transport	-	2
GO:0006814 sodium ion transport	-	1
GO:0006820 anion transport	1	2
GO:0006821 chloride transport	1	1
GO:0006869 lipid transport	1	1
GO:0006886 intracellular protein transport	1	1
GO:0006915 apoptotic process	-	1

GO:0006940 regulation of smooth muscle contraction	1	-
GO:0006959 humoral immune response	1	-
GO:0007010 cytoskeleton organization	2	-
GO:0007015 actin filament organization	-	1
GO:0007017 microtubule-based process	-	1
GO:0007018 microtubule-based movement	1	-
GO:0007165 signal transduction	7	5
GO:0007166 cell surface receptor signaling pathway	-	2
GO:0007169 transmembrane receptor protein tyrosine kinase signaling	1	-
GO:0007186 G-protein coupled receptor signaling pathway	6	6
GO:0007189 adenylate cyclase-activating G-protein coupled receptor	1	1
GO:0007205 protein kinase C-activating G-protein coupled receptor	-	1
GO:0007264 small GTPase mediated signal transduction	5	2
GO:0007275 multicellular organism development	-	1
GO:0007399 nervous system development	1	1
GO:0007596 blood coagulation	1	-
GO:0007601 visual perception	-	1
GO:0008033 tRNA processing	1	-
GO:0008152 metabolic process	1	-
GO:0008299 isoprenoid biosynthetic process	1	-
GO:0008544 epidermis development	-	1
GO:0008616 queuosine biosynthetic process	1	-
GO:0009058 biosynthetic process	1	-
GO:0009060 aerobic respiration	-	1
GO:0009072 aromatic amino acid family metabolic process	-	1
GO:0009190 cyclic nucleotide biosynthetic process	1	1
GO:0009247 glycolipid biosynthetic process	1	-
GO:0009451 RNA modification	1	-
GO:0010579 positive regulation of adenylate cyclase activity	-	1
GO:0015031 protein transport	1	1
GO:0015937 coenzyme A biosynthetic process	-	1

GO:0015992 proton transport	1	-
GO:0016192 vesicle-mediated transport	1	-
GO:0016567 protein ubiquitination	1	-
GO:0016568 chromatin modification	1	-
GO:0016573 histone acetylation	1	-
GO:0016579 protein deubiquitination	2	2
GO:0016598 protein arginylation	-	1
GO:0019229 regulation of vasoconstriction	1	-
GO:0023052 signaling	-	1
GO:0030150 protein import into mitochondrial matrix	1	-
GO:0030168 platelet activation	1	-
GO:0032259 methylation	-	1
GO:0035023 regulation of Rho protein signal transduction	1	-
GO:0035556 intracellular signal transduction	2	2
GO:0042157 lipoprotein metabolic process	1	1
GO:0042311 vasodilation	-	1
GO:0042981 regulation of apoptotic process	1	2
GO:0043039 tRNA aminoacylation	2	-
GO:0043066 negative regulation of apoptotic process	1	-
GO:0043248 proteasome assembly	-	1
GO:0045823 positive regulation of heart contraction	1	1
GO:0046907 intracellular transport	-	1
GO:0051260 protein homooligomerization	-	2
GO:0055085 transmembrane transport	1	5
GO:0055114 oxidation-reduction process	5	5
GO:0071157 negative regulation of cell cycle arrest	1	1
Total GO: 106	79 (75%)	62 (58%)
Total proteins:	126	115

SUPPL TABLE S8 Proteins for which InterPro and GO database categories have not been attributed*.

Protein	Gene (Gene ID)	HFs	BCPs	Brief description of the protein

A8MU76.2	NPAP1L (729159)	+	+	NPAP1L nuclear pore associated protein 1 like
A8MUU9.3		+		Uncharacterized protein
A8MV72.2	LOC100132229 (100132229)		+	LOC100132229 nuclear pore associated protein 1 pseudogene
A8MX80.2	LOC100129307 (100129307)	+	+	LOC100129307 putative UPF0607 protein ENSP00000383144
E9PAV3.1	NACA (4666)		+	NACA nascent polypeptide associated complex subunit alpha
Q5T8R8.1	C9orf66 (157983)	+		C9orf66 chromosome 9 open reading frame 66
Q8NBZ0.1	INO80E (283899)	+	+	INO80E INO80 complex subunit E
Q8NEA5.1	C19orf18 (147685)	+		C19orf18 chromosome 19 open reading frame 18
Q96C57.2	C12orf43 (64897)		+	C12orf43 chromosome 12 open reading frame 43
Q96HJ3.2	CCDC34 (91057)	+		CCDC34 coiled-coil domain containing 34
Q96NF6.1	C8orf49 (606553)	+		C8orf49 chromosome 8 open reading frame 49 (putative)
Q9P0P8.1	C6orf203 (51250)		+	C6orf203 chromosome 6 open reading frame 203

*Protein code according to UniProtKB/Swiss-Prot.
